# Structural interventions that affect racial inequities and their impact on population health outcomes: a systematic review

**DOI:** 10.1186/s12889-022-14603-w

**Published:** 2022-11-24

**Authors:** Emily C. Clark, Emily Cranston, Tionné Polin, Sume Ndumbe-Eyoh, Danielle MacDonald, Claire Betker, Maureen Dobbins

**Affiliations:** 1 National Collaborating Centre for Methods and Tools, McMaster University, McMaster Innovation Park, 175 Longwood Rd S, Suite 210a, Hamilton, ON L8P 0A1 Canada; 2 Dalla Lana School of Public Health, University of Toronto, 155 College Street, 6th Floor, Toronto, ON M5T 3M7 Canada; 3 National Collaborating Centre for Determinants of Health, St. Francis Xavier University, 2400 Camden Hall, Antigonish, NS B2G 2W5 Canada; 4 School of Nursing, McMaster University, Health Sciences Centre, 2J20, 1280 Main St W, Hamilton, ON L8S 4K1 Canada

**Keywords:** Systematic review, Racism, Structural racism, Health inequity, Policy, Health outcome, Determinants of health, Race, Ethnicity

## Abstract

Structural racism is the historical and ongoing reinforcement of racism within society due to discriminatory systems and inequitable distribution of key resources. Racism, embedded within institutional structures, processes and values, perpetuates historical injustices and restricts access to structural factors that directly impact health, such as housing, education and employment. Due to the complex and pervasive nature of structural racism, interventions that act at the structural level, rather than the individual level, are necessary to improve racial health equity. This systematic review was conducted to evaluate the effects of structural-level interventions on determinants of health and health outcomes for racialized populations. A total of 29 articles are included in this review, analyzing interventions such as supplemental income programs, minimum wage policies, nutrition safeguard programs, immigration-related policies, and reproductive and family-based policies. Most studies were quasi-experimental or natural experiments. Findings of studies were largely mixed, although there were clear benefits to policies that improve socioeconomic status and opportunities, and demonstrable harms from policies that restrict access to abortion or immigration. Overall, research on the effects of structural-level interventions to address health inequities is lacking, and the evidence base would benefit from well-designed studies on upstream policy interventions that affect the structural determinants of health and health inequities and improve daily living conditions.

**Supplementary Information:**

The online version contains supplementary material available at 10.1186/s12889-022-14603-w.

## Background

Racism is a system of power that manifests at all levels of society, resulting in the differential treatment and access to resources and opportunities based on one’s racial identity [1, 2]. Racial health inequities, power imbalances, and injustices act on institutional and structural levels. Jones’ framework, Levels of Racism, aligns with this perspective and purports that racism operates at three levels: institutionalized, personally mediated, and internalized [3]. Of these three, the institutional, or structural, level is the most fundamental and the necessary starting point for meaningful change [3]. Structural racism refers to the historical and ongoing pervasive reinforcement of racism within society and its interconnected, codependent institutions [4]. Specifically, structural racism is how populations are disadvantaged due to discriminatory systems and inequitable distribution of key resources at every level of government and within every sector of society [4]. At the structural level, racism is entrenched within institutional structures, processes and values, perpetuating historical injustices and restricting access to housing, education, employment and health and social services [5]. For example, the “redlining” to deny mortgage loans in predominantly Black neighbourhoods, preventing the accumulation of real estate wealth and perpetuation of wealth inequity [6–8]. Schools within redlined neighbourhoods receive fewer resources resulting in long-term educational inequities [4, 9, 10]. Developers were less likely to invest in redlined neighbourhoods, leading to reduced access to healthy groceries and healthcare facilities [11–13]. This ultimately created a pathway between racism and health, due to more proximal outcomes such as social deprivation and economic injustices [4]. Similarly, Williams and Mohammed’s Framework for the Study of Racism on Health outlines interconnected pathways, where basic causes (e.g., structural racism, institutions) and one’s social status (e.g., gender, socioeconomic status, race), influence proximal pathways (e.g., opportunities, resources, psychosocial stressors), which result in various responses and behaviours that can lead to a spectrum of negative population health outcomes [14].

Racism affords privileges for individuals and groups deemed superior based on actual or perceived proximity to Whiteness and leads to explicit and implicit maltreatment and disenfranchisement of those deemed inferior [5]. Critical Race Theory positions race as a social construct and outlines the interplay between race and racism, the means of power and domination [15]. Critical Race Theory seeks to eliminate the racial imbalance of power through the analysis of the pervasiveness of racism and its influence on society [15, 16] . Structural determinism, a key tenet of Critical Race Theory [1] posits that macro-level forces have a crucial role in creating and maintaining inequities and that racism and intersecting systems of power function to preserve the power of the dominant group [1]. The application of Critical Race Theory can improve public health policy and practice as it encourages researchers, practitioners, and policymakers to act upstream with interventions that impact the root causes of racialized inequities [1]. A key element of this work is the need to disrupt White supremacy and acknowledge the pervasiveness of Whiteness through all levels and settings within society [17, 18].

There is an extensive body of research demonstrating that structural racism is an important determinant of health [19]. Structural racism impacts health directly as well as through persistent racial income inequities and socioeconomic status, which are known to drive racial health inequities. Data from the 2017 Pan-Canadian Health Inequalities data tool found the prevalence of diabetes in Black people in Canada was 2.1 times higher than in their White counterparts; similarly, Black people are more likely to report having fair or poor health [20]. Indigenous populations in Canada endure disproportionately higher rates of infant mortality, tuberculosis, obesity, diabetes, youth suicide and environmental contaminants, resulting from ongoing colonialism and racism [21].

Despite the role of structural racism in health outcomes, there are limited studies that evaluate structural racism and related interventions [22, 23]. Research is largely focused on individual and interpersonal racism, rather than how racism embedded in systems at the structural level affects racial health inequities and suboptimal health outcomes [23]. Consequently, there is a need to measure the impacts of racism at not only the individual level, but at the structural levels [24]. Upstream interventions, which extend beyond those at the level of proximal outcomes, are necessary to improve population health inequities [14]. These include interventions that change the social, physical, economic or political environments that influence health, such as economic stability, educational and employment opportunities, discrimination and racism, access to healthy food and healthcare [25, 26].

Previous literature reviews have highlighted examples of structural interventions that have affected racial health inequities [4, 25]. Bailey et al, 2017, provides a comprehensive overview of the pathways through which structural racism can affect population health and cites several examples of interventions designed to reduce racial health inequities, but does not include a systematic search or outcome data for these studies [4]. Similarly, Brown et al, 2019, thoroughly discusses the challenges and opportunities for structural interventions to reduce racial health inequities and includes several examples of policy interventions to improve health, but again did not systematically search for studies or include outcome data [25]. These reviews help highlight the causal links between structural interventions and racial health equity, but without health outcome data it is not possible to quantify their impact for racialized populations. There are examples of systematic reviews that have been conducted to evaluate structural interventions that may affect health equity, but these have been limited to one policy domain and often do not report data for health outcomes stratified by race [27–31]. By limiting analysis to one policy domain, it is not possible to determine the relative potential influence of different policy domains on racial health equity. At this time, a systematic review of the literature evaluating the effect of structural interventions on racial health equity that reports health outcomes stratified by raceis needed to help inform future policy development.

**Fig. 1:**
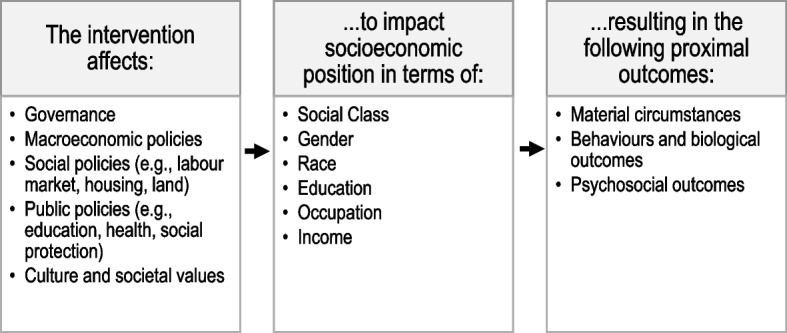
Adapted framework to assess impact of intervention on structural determinants of health. Based on the World Health Organization (WHO) Commission on Social Determinants of Health (CSDH) conceptual framework [33], included studies evaluated interventions that affect structural policies to impact socioeconomic position, to effect change in intermediary determinants of health, such as material circumstances, behaviors or biological outcomes and psychosocial outcomes

This systematic review was conducted alongside a larger scoping review on interventions to address structural determinants that affect population health outcomes, with a narrower focus on racial health inequities, to inform policy development in Canada. For the scoping review, structural determinants of health were defined according to the Pan American Health Organization (PAHO) Equity Commission’s Conceptual Framework for Structural Drivers of Health Equity and Dignified Life [32] and the World Health Organization’s Commission on Social Determinants of Health (CSDH) conceptual framework (Fig. 1) [33]. According to this framework, structural drivers, which include governance, economic policies, social policies, public policies and cultural or societal values, influence an individual’s status within the social hierarchy [33]. Social hierarchies systematically allocate unequal power and resources according to the socioeconomic and political context, resulting in inequity [33]. This systematic review is novel in its investigation of structural level interventions for any policy type that affect structural drivers through the socioeconomic and political context to affect inequities in racial health outcomes.

## Methods

### Protocol and registration

The review protocol was registered with the International Prospective Register of Systematic Reviews (PROSPERO; Registration #CRD42021266334). The review was conducted and reported following the Preferred Reporting Items for Systematic Reviews and Meta-Analyses (PRISMA) statement for reporting systematic reviews and meta-analyses (Moher, 2009).

### Eligibility criteria

Published and unpublished (grey literature) studies in English or French were eligible for inclusion. Review papers, such as literature and systematic reviews, were excluded.

#### Population

Study populations included those affected by systemic racism and racial health inequities, such as Indigenous and Black populations and other racialized groups (e.g., Latinx, Asian, etc.). The term “racialized” is used to describe members of populations affected by systemic racism, rather than terms such as “marginalized” or “vulnerable”, as these terms have been rejected by members of these populations for suggesting exclusion or deficit [34–36]. In this review, the term “racialized” does not include individuals who are or identify as White as they are not disadvantaged by the discriminatory systems of power that depend on the social construction of race in society. Since this review was completed to inform policy development in Canada, Only studies conducted in the 38 member countries of the Organization for Economic Co-operation and Development (OECD) were included in this analysis to best align with Canada’s political and economic context and to better inform policy development within Canada [37]. This list of countries is broader than the Group of Twenty (G20), but still limits analysis to contexts that align with Canada’s democratic and high-income context.

#### Intervention

Studies of randomized or non-randomized structural-level interventions that measure health outcomes were included. For this review, included interventions aligned with the World Health Organization (WHO) Commission on Social Determinants of Health (CSDH) conceptual framework [33]. For example, this included interventions such as structural policies that impact socioeconomic position, and thus effect change in intermediary determinants of health, such as material circumstances, behaviors or biological outcomes and psychosocial outcomes (Fig. 1). Studies on private health insurance and Medicaid were excluded as these have limited relevance to the Canadian context.

#### Comparator

Studies that compared the effect of an intervention to a control group or before and after implementation were included.

#### Outcome

Studies must have reported population or public health outcomes, such as mortality and morbidity, physical and mental health and health behaviours for a racialized population or stratified by racial identity.

### Information sources and search strategy

The larger scoping review was undertaken by the National Collaborating Centre for Determinants of Health (NCCDH) to inform an environmental scan of public health in Canada. The review was registered with the Open Science Framework registry (https://osf.io/dyn93). The NCCDH conducts periodic environmental scans to assess how the centre can support public health to advance health equity. The research question addressed in the scoping review was “What is known about interventions for the structural determinants of health and health inequity as they affect population and public health outcomes in G20 and Nordic countries?”

The studies in this systematic review are a subset of those identified for the above related scoping review. The search strategy was developed and conducted by a librarian with expertise in scoping and systematic reviews. The scope of the review was broad, without one single outcome, population, or intervention.

The initial search strategy was designed for Medline, then adapted to other sources, such as Google Scholar. EBSCO Medline was searched for publications from 2005, combining key terms for racialized populations, determinants of health and health outcomes, and government policies or interventions. A full search strategy is included in Additional file 1. To capture grey literature, Google Scholar was searched from 2015 as it indexes scholarly, professional and pre-print literature across many disciplines, including institutional repositories, government reports, academic dissertations, book chapters, conference abstracts and court opinions. The Google Scholar search was limited to 2015 so that the number of retrieved references was manageable. Overall, a smaller set of databases that were most likely to include relevant results were searched with very broad search terms, to be as comprehensive as possible while remaining feasible. The initial search for literature was conducted in October 2020, and then updated on October 11, 2022. Studies captured in the updated search have been incorporated in this review.

Citation tracking was also conducted to locate literature relevant to articles selected for full-text review, using the Google Scholar “Cited by” feature.

Retrieved references were imported into the Rayyan Intelligent Systematic Review platform for de-duplication and screening by title and abstract. References selected for full-text screening were imported into the Covidence systematic review platform. All full-text screening was performed independently by two reviewers (EC, TP). Discrepancies were resolved by discussion, and if no consensus was reached, were resolved by discussion with a third reviewer (ECC).

### Data extraction

Data extraction was conducted in Covidence. Characteristics of the study, design, intervention, population, and outcomes were extracted. Study results as they related to racial disparities were also extracted. Statistical significance was considered at *p* < 0.05, regardless of the threshold defined in the study.

Two independent reviewers (EC, TP) completed data extraction with guidance from a third reviewer (SNE). Extracted data were checked by a fourth reviewer who resolved any discrepancies (ECC).

### Quality assessment

Non-randomized studies of interventions and natural experiments were evaluated for methodological quality using the Joanna Briggs Institute (JBI) Checklist for Quasi-Experimental Studies. Randomized controlled trials (RCTs) were evaluated for methodological quality using the Joanna Briggs Institute (JBI) Checklist for Randomized Controlled Trials. Checklist scores were labelled based on the proportion of possible criteria met by the study, with Low if 50% or less possible criteria were met, Moderate for 50 to 75% and High for over 75%. All studies were critically appraised by two independent reviewers (EC, TP). A third reviewer checked results and resolved discrepancies (ECC).

### Data analysis

It was expected that given the broad inclusion of studies with interventions from different policy domains there would be high heterogeneity between studies, so a meta-analysis of results was not planned. Study characteristics and results were synthesized narratively with a focus on the impact of individual study quality. Studies were grouped and analyzed by intervention policy domains. Analysis was guided by the Pan American Health Organization (PAHO) Equity Commission’s Conceptual Framework for Structural Drivers of Health Equity and Dignified Life [32], as it focuses on the impact of racism and colonization. Vote counting based on the direction of effect was used to determine whether most studies found a positive or negative effect [38].

## Results

**Fig. 2:**
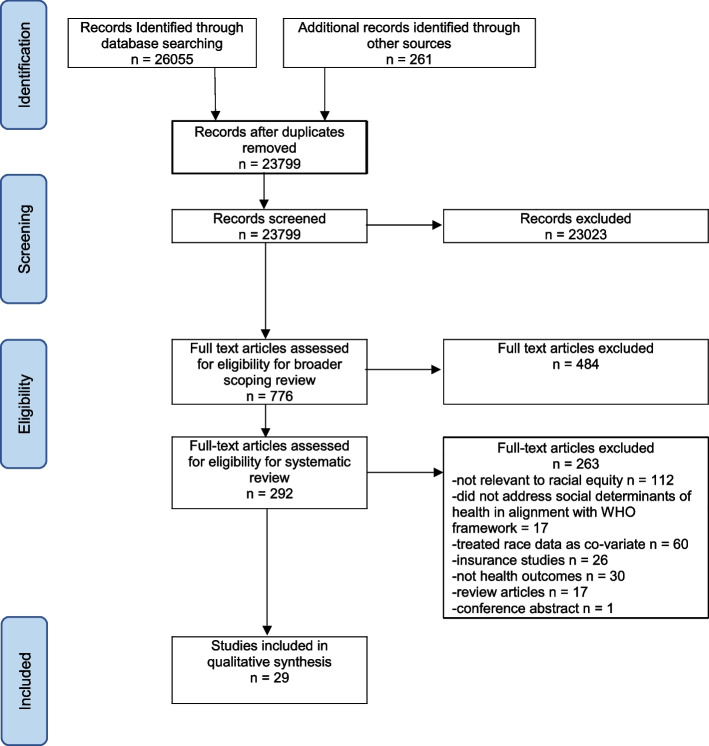
PRISMA Flow chart

Database searching for the broader, related scoping review retrieved 24,311 records and citation tracking retrieved an additional 261, for a total of 26,055 records. After removing duplicates, 23,799 records were screened by title and abstract, resulting in 776 records for full text review for inclusion in the broader scoping review. Of those 776 records, 292 articles were screened for health outcomes by race for eligibility for this systematic review, resulting in 29 included articles. Studies excluded at this stage are listed in Additional file 2. See Fig. 2 for a PRISMA flow chart illustrating the article search and selection process.

### Study characteristics

Characteristics of included studies are summarized in Table 1. Of the 29 included studies, 28 were natural or quasi-experiments and one was a randomized controlled trial. Most were conducted in the USA [39–55, 57–63, 67], along with one study in Canada [65] and two in Australia [64, 66]. Studies were published from 2008 to 2021, with data collection periods starting as early as 1927.

**Table 1:** Characteristics and Findings of Included Studies

Author, Year	Study Design	Setting	Intervention	Sample size, population	Data Collection Period and Source	Racial or Ethnic Identities	Comparator	Outcome	Findings	Quality
Financial Policies										
Averett, 2012 [39]	Quasi-experimental	USA	Expansion of Earned Income Tax Credit (EITC)	*N* = 12,686﻿, Low-to-moderate income mothers	1992–1998, National Longitudinal Survey of Youth 1979 Cohort	Stratified analysis: Black, Hispanic, White	Before and after expansion of EITC	Smoking status	No statistically significant difference in smoking status for Black or Hispanic mothers.	Moderate
Balan-Cohen, 2008 [40]	Quasi-experimental	USA	Old Age Assistance (OAA)	Total sample size NR; low-income seniors	1927–1955; Vital Statistics Reports	Stratified analysis: Black, White	Non-recipients of OAA	Mortality from treatable illnesses, behavioral causes and cardiovascular disease	Reduced mortality for Black (12%) and White (17%) in non-Southern States. Statistical difference not evaluated. No statistically significant difference in mortality in Southern States.	Moderate
Braga, 2020 [41]	Quasi-experimental	USA	Earned Income Tax Credit during childhood	*N* = 2393, children of low-to-moderate income workers	1968–2017, Panel Study of Income Dynamics	Stratified analysis: Black, Hispanic, other races, White	Differing EITC levels across States	Self-reported overall health, obesity, high blood pressure, functional limitations, emotional problems during adulthood	No statistically significant differences in self-reported health outcomes for Black, Hispanic or other races for different EITC levels.	Moderate
Bruckner, 2013 [42]	Quasi-experimental	USA	Earned Income Tax Credit	*N* = 259,480﻿, low-to-moderate income pregnant mothers	1989–1997, California Birth Files	Stratified analysis: Black, White	Non-recipients of EITC	Very low birthweight (< 1500 g) among live singleton births	Odds of very low birthweight increased for Black women when EITC received within 2 months of delivery (OR 1.31, 95%CI 1.09, 1.58). No statistically significant differences for White women or when EITC received earlier in pregnancy.	Moderate
Cloud, 2019 [43]	Quasi-experimental	USA	State-level minimum wage policies	*N* = 73 Metropolitan Statistical Areas, each with population > 500,000 residents	2007–2015, CDC HIV name-based reporting system and USA Census Bureau	Primary population: Black	Before and after minimum wage increase	Annual incidence of HIV diagnoses in heterosexual individuals	Metropolitan Statistical Areas with a $1.00 higher minimum wage had a 27.12% (95% CI 18.06, 35.18) lower rate of incident HIV cases in heterosexual Black individuals.	Moderate
Goldstein, 2020 [44]	Quasi-experimental	USA	Government expenditure on non-healthcare services	Total sample size NR; infants and mothers	2000–2016, US National Center for Health Statistics	Stratified analysis: Black, Hispanic, Asian, White	Differing levels of state and local government expenditures across States	Infant mortality	No significant difference in the change in infant mortality per increase in government spending for infants born to mothers of different races.	Moderate
Hoynes, 2015 [45]	Quasi-experimental	USA	Earned Income Tax Credit	*N* = 17,865,﻿ low-to-moderate income pregnant women	1983–1999, Vital Statistics Reports	Stratified analysis: Black, Hispanic, White	Before and after expansion of EITC	Birthweight, low birthweight (< 2500 g)	Reduction in low birthweight incidence for Black women (0.73%, *p* < 0.01).	Moderate
Jagannathan, 2010 [46]	RCT	USA	New Jersey Family Development Program (FDP) Welfare Reform	*N* = 8393﻿, low-income women	1992–1996; primary data	Stratified analysis: Black, Hispanic, White	Welfare recipients not subject to FDP reform	Clinically diagnosed depressive or anxiety disorders	For Black women subject to FDP reform, decreased incidence of anxiety disorder diagnosis (−15.3%, *p* < 0.05) and depressive disorder diagnosis (− 2.1%, *p* < 0.05). For Hispanic women subject to FDP reform, increased incidence of depressive disorder diagnosis (68%, *p* < 0.05)	Moderate
Komro, 2019 [47]	Quasi-experimental	USA	Earned Income Tax Credit	*N* = 29,269,997,﻿ low-to-moderate income pregnant women	1994–2013, Vital Statistics Reports	Stratified analysis: Black, Hispanic, White	Non-recipients of EITC, differing EITC levels across States, EITC with and without refund	Birthweight, low birthweight (< 2500 g), weeks gestation	Increase in birth weight (16.12–37.16 g, *p* < 0.01), reduction in incidence of low birthweight (0.6–1.4%, *p* = 0.0001) and increase in gestational age (0.38–0.46%, p for Black women when receiving EITC. The relative percent changes are not significantly different for White women.	High
Rosenquist, 2019 [48]	Quasi-experimental	USA	State-level Minimum wage policies	*N* = 3,869,884, low-income	1980–2010, Vital Statistics Reports	Stratified analysis: Black, White	Different minimum wage levels across States	Infant mortality	Decreased odds of infant mortality for Black women in states with higher minimum wage (adjusted OR 0.80, 95%CI 0.68, 0.94), and in states with greatest increase in minimum wage (adjusted OR 0.89, 95% CI 0.82, 0.96).	Moderate
Nutrition Safeguards										
Arons, 2016 [49]	Quasi-experimental	USA	Special supplemental nutrition program for women, infants, and children (WIC)	*N* = 327 mother-child dyads	2006–2011, primary data	Stratified analysis: Black	Full sample	Childhood socioemotional development	No significant difference in socioemotional development in Black children whose mothers receive WIC.	Moderate
Booshehri, 2021 [50]	Quasi-experimental	USA	Special Supplemental Nutrition Program (SNAP) eligibility at 60 years of age	*N* = 15,980, low-income older adults (age 60–64 years)	2008–2013, Medical Expenditure Panel Survey	Stratified analysis: Black, Hispanic, other races, White	Adults aged 56–59 not yet eligible for SNAP	Diet-related morbidities	Upon reaching SNAP eligibility at age 60, decreased prevalence of hypertension (−13.95%, *p* < 0.01) for Black individuals, decreased prevalence of angina (−6.94%, *p* < 0.01) and stroke (−4.48%, *p* < 0.01) for Hispanic individuals.	Moderate
Conrad, 2017 [51]	Quasi-experimental	USA	Special Supplemental Nutrition Program (SNAP)	*N* = 499,741	2000–2011, National Health Interview Survey	Stratified analysis: Black, Hispanic	SNAP-eligible non-participants	All-cause mortality, cardiovascular mortality	Compared to SNAP-eligible non-participants of same race, statistically significant (*p* < 0.01) higher risk of all-cause and diabetes-related mortality for Black SNAP participants; higher all-cause mortality for Hispanic SNAP participants; higher all-cause, cardiovascular and diabetes-related mortality for White SNAP participants.	Low
Jia, 2020 [52]	Quasi-experimental	USA	Updated National School Lunch Program (NSLP)	*N* = 9172, children	2005–2016, National HEalth and Nutrition Examination Survey (NHANES)	Stratified analysis: Black, Hispanic	Before and after update to NSLP	Dietary intake	Black students increased fruit and vegetable intake by 0.27 cups (95% CI = 0.07, 0.46). Hispanic students reduced in weekday fruit and vegetable intake by 0.29 cups (95% CI = -0.50, −0.08).	Moderate
Kong, 2014 [53]	Quasi-experimental	USA	Revision to Special supplemental nutrition program for women, infants, and children (WIC) to provide more whole grains, fruits, vegetables, and fewer foods with high saturated fat content	*N* = 295, parent-child dyads	2009–2011, primary data	Stratified analysis: Black, Hispanic	Before and after revisions to WIC	Dietary intake, food group intake, diet quality	No significant changes in nutrient intake and overall diet quality were observed for mothers. Black children increased consumption of sugar-sweetened beverages (*p* = 0.01). Hispanic children improved in diet quality (*p* = 0.02) and saturated fat intake (*p* = 0.0004).	Moderate
Immigration										
Bruzelius, 2019 [54]	Quasi-experimental	USA	National anti-immigration policy changes, increased ICE arrest rates	*N* = 118,883, adults	2014–2018, Behavioral Risk Factor Surveillance System (BRFSS)	Primary population: Hispanic or Latinx of any race	Before and after immigration policy changes	Mental health	No significant changes in reports of at least one poor mental health day in preceding month, any indication of poor mental health or reports of frequent mental distress.	High
Hamilton, 2020 [55]	Quasi-experimental	USA	Deferred Action for Childhood Arrivals (DACA)	*N* = 72,613, singleton births	2010–2014, National Centre for Health Statistics	Primary population: Hispanic or Latinx of any race	Infants born to DACA-ineligible mothers; before and after DACA enactment	Birthweight, gestational age	For infants born to DACA-eligible mothers and conceived after DACA enactment, • Low birthweight decreased by 1% (*p* < 0.05) • Very low birthweight decreased by 0.4% (*p* < 0.05) • Birthweight increased by average 28.8 g (*p* < 0.01) • Average gestational age increased by 0.09 weeks (*p* < 0.05)	Moderate
Hatzenbuehler, 2017 [56]	Quasi-experimental	USA	State-level exclusionary immigration policies	*N* = 293,081, adults	2012, Behavioral Risk Factor Surveillance System (BRFSS)	Primary population: Hispanic or Latinx of any race	States with less exclusionary immigration policies	Mental health	In states with more exclusionary immigration policies, Latinx had 1.14 times (95% CI 1.04,1.25) the rate of poor mental health days than Latinx in states with less exclusionary immigration policies. The association between state immigration policies and rate of poor mental health days higher for Latins than non-Latinx (RR 1.03, 95% CI = 1.01, 1.06).	Moderate
Potochnick, 2017 [57]	Quasi-experimental	USA	Federal 287(g) program increasing immigration policy enforcement	*N* = 58,353, adults	2004–2009, Current Population Survey Food Supplement Survey (CPS-FSS)	Primary population: Hispanic or Latinx of any race	Before and after 287(g) enactment	Food security	Enactment of 287(g) was associated with 10.9% (*p* < 0.01) increase in food insecurity for Mexican non-citizen households with children. No significant effects for Hispanic citizen, non-Hispanic White or non-Hispanic Black households.	Moderate
Torche, 2019 [58]	Quasi-experimental	USA	Arizona’s Senate Bill SB1070 increasing immigration policy enforcement	*N* = 1.5 million, mother-child dyads	2007–2012, Centers for Disease Control and Prevention and Arizona Department of Health Services	Primary population: Hispanic or Latinx of any race	Before and after enactment of SB1070	Birthweight, gestational age, birth rate	Following enactment of SB1070, significant decline in birthweight (15 g, *p* < 0.01) for infants born in late 2010 to immigrant Latina mothers exposed to passage of law during pregnancy.	High
Vargas, 2017 [59]	Quasi-experimental	USA	State-level exclusionary immigration policies	*N* = 1200, adults	2011, Latino Decisions/ImpreMedia Survey	Primary population: Hispanic or Latinx of any race	States with less punitive anti-immigration policies	Overall health status	Compared to Latinx respondents in states with more punitive anti-immigration policies, Latinx respondents in states with low or medium punitive anti-immigration laws were more likely to report optimal health (OR 1.8, p ≤ 0.05 and OR 1.5, p ≤ 0.05 for low and medium punitive laws, respectively)	Moderate
Venkataramani, 2017 [60]	Quasi-experimental	USA	Deferred Action for Childhood Arrivals (DACA)	*N* = 14,973, adults	2008–2015, US National Health Interview Survey (NHIS)	Primary population: Hispanic or Latinx of any race	DACA-ineligible adults	Overall health, psychological distress	Implementation of DACA associated with significant reductions in psychological distress scores (incident RR 0.78, 95% CI = 0.56, 0.95) and odds of reporting moderate or worse psychological distress (adjusted incident RR 0.62, 95% CI = 0.41, 0.93) for DACA-eligible compared to DACA-ineligible respondents.	Moderate
Family and Reproductive Policies										
Coles, 2010 [61]	Quasi-experimental	USA	Restrictive Abortion Statutes (parental involvement laws, Medicaid funding restrictions, mandatory waiting periods)	*N* = 8245, adolescents aged 10–17 years	2000–2005, Pregnancy Risk Assessment Monitoring System (PRAMS)	Stratified analysis: Black, Hispanic	States with less restrictive abortion policies	Unwanted or mistimed births. (Mistimed births were reported in cases where the mother wanted pregnancy only later in life)	In states with Medicaid funding restrictions, Black minors had higher rates of mistimed (RR 4.11, *p* < 0.05) compared to states with no such restrictions.	Low
Hamad, 2019 [62]	Quasi-experimental	USA	Paid family leave policies	*N* = 306,266, post partum women	2003–2015, National Immunization Survey	Stratified analysis: Black, Hispanic	Before and after implementation of paid family leave policies	Self-reported breastfeeding at any time and at 3-, 6- and 12-months post-partum	Following implementation of paid family leave policies, Hispanic mothers were 2.3% more likely to report exclusive breastfeeding at 6 months (95% CI = 1.2, 3.4). Black mothers were 3.8% less likely to report breastfeeding at 6 months (95% CI = -7.3, −0.3), 2.9% less likely at 12 months (95% CI = -4.8, −1.0) and reported shorter breastfeeding duration by 15.5 days (95% CI = -24.2, −6.9).	Moderate
Sudhinaraset, 2020 [63]	Quasi-experimental	USA	Reproductive rights policies	*N* = 3,945,875, women	2014–2015, National Center for Health Statistics	Stratified analysis: Black, Hispanic, Asian	States with less restrictive abortion policies	Birthweight, preterm birth	In states with the least restrictive rights policies, Black women had a lower risk of low birth weight than Black women in the most restrictive states (Adjusted RR 0.92, 95% CI = 0.86, 0.99)	Moderate
Policies for Indigenous Populations										
Clough, 2017 [64]	Quasi-experimental	Australia	Alcohol Management Plans (AMPs)	*N* = 1211, adults in Queensland’s remote Indigenous communities	2014–2015, primary data	Primary population: Aboriginal and Torres Strait Islanders	Before and after implementation of AMPs	“Favourable” and “unfavourable” alcohol-related health outcomes in the community	Majority of respondents agreed that AMPs made communities safer and less violent. Majority of respondents reported more cannabis use and binge drinking, that alcohol was no less available, and that more law enforcement activities such as fines and arrests occurred.	Moderate
Feir, 2015 [65]	Quasi-experimental	Canada	Residential school system	*N* = 4939, First Nations, Métis or Inuit children	2001, Aboriginal Peoples Survey of Children and Youth (APSCY)	Primary population: First Nations, Métis or Inuit children	Children whose mothers did not attend residential schools	BMI, height, birthweight, childhood injuries	Children whose mothers attended residential school had higher average BMI (*p* < 0.05) than children whose mothers did not attend residential school.	Moderate
Larson, 2019 [66]	Quasi-experimental	Australia	Indigenous land and sea management programs (ILSMPs)	*N* = 190 Ewamian people, Nyikina Mangala, Bunuba people and Walmajarri people	Time period NR, primary data	Primary population: Aboriginal and Torres Strait Islanders	Before and after implementation of ILSMPs	Holistic wellbeing impact evaluation (W-IE)	After implementation of ILSMPs, respondents reported positive change in satisfaction for following wellbeing factors • “Country looked after” (health of land) • Information and communications technology • Legal right to country • Business ownership A negative change in satisfaction was reported for consuming traditional foods.	Low
Environmental										
Furzer, 2020 [67]	Quasi-experimental	USA	Environmental Policy: PM 2.5 and Clean Air Act Regulations	Not applicable	2000–2016, primary data and 2020, Johns Hopkins University Coronavirus Resource Center	Primary population: Black, minority	Regions without air pollution limit attainment	COVID-19 deaths	Counties with high proportion of Black or minority residents are less likely to attain air pollution limits, with 6.8–16% more COVID-19 deaths for Black or minority residents.	Moderate

*CI* Confidence Interval, *OR* Odds Ratio, *RR* Risk ratio, *NR* Not reported, *BMI* Body Mass Index, *PM* Particulate Matter

Interventions were targeted at several major policy domains, including financial (*n* = 10) [39–48], nutrition safeguards (*n* = 5) [49–53], immigration (*n* = 7) [54–60], family and reproductive rights (*n* = 3) [61–63], policies for Indigenous populations (*n* = 3) [64–66] and environment (*n* = 1) [67]. Only the interventions in the immigration policy domain and those related to Indigenous populations were explicitly designed to affect racial inequities. All other interventions were targeted to low-income or general populations, but reported outcomes stratified by race.

Twelve studies were specifically designed to evaluate health outcomes for racial or ethnic populations [43, 54–58, 60, 64–67]. The remaining 17 studies evaluated racial and/or ethnic differences as a secondary objective or as part of a stratified analysis [39–42, 44–53, 61–63], including all except one study of financial interventions [43], all studies of nutrition safeguards and all studies of family and reproductive rights policy interventions. Studies included White, Black, Latinx, Hispanic, Asian and Indigenous populations. Approximately one-third (*n* = 11) [39, 43, 45, 52–54, 57, 58, 62, 64, 66] of the included studies compared outcomes before and after policy implementation, while the remaining (*n* = 18) [40–42, 44, 46–51, 55, 56, 59–61, 63, 65, 67] compared outcomes for similar populations not subject to the policy or differing levels of policy implementation.

### Quality of included studies

Most studies were rated as being of moderate methodological quality (*n* = 23), with three each rated as high and low quality. Quality appraisal results for the 28 quasi-experimental studies are included in Table 2 and for the randomized controlled trial in Table 3.

**Table 2:** Quality Assessment of Included Quasi-Experimental Studies

Study	JBI Checklist for Quasi-Experimental Studies										
	1. Clear cause and effect	2. Similar participants in comparisons	3. Participants received similar treatment	4. Control group	5. Multiple outcome measurements	6. Follow up complete or differences described	7. Outcomes measured in same way	8. Reliable outcome measurement	9. Appropriate statistical analysis	Overall score^a^/Possible score	Rating^b^
Arons, 2016 [49]	Yes	Unclear	No	No	Yes	N/A	Yes	Yes	Yes	5/8	Moderate
Averett, 2012 [39]	Yes	Unclear	No	Yes	Yes	N/A	Yes	Yes	Yes	6/8	Moderate
Balan-Cohen, 2009 [40]	Yes	Unclear	No	No	Yes	N/A	Yes	Yes	Yes	5/8	Moderate
Booshehri, 2021 [50]	Yes	No	No	Yes	Yes	N/A	Yes	Yes	Yes	6/8	Moderate
Braga, 2020 [41]	Yes	No	No	Yes	Yes	N/A	Yes	Yes	Yes	6/8	Moderate
Bruckner, 2013 [42]	Yes	Unclear	No	Yes	Yes	N/A	Yes	Yes	Yes	6/8	Moderate
Bruzelius, 2019 [54]	Yes	N/A	Yes	No	Yes	N/A	Yes	Yes	Yes	6/6	High
Cloud, 2019 [64]	Yes	Unclear	Unclear	No	Yes	N/A	Yes	Yes	Yes	5/8	Moderate
Clough, 2017 [43]	Yes	N/A	N/A	No	No	No	Yes	Yes	Yes	4/7	Moderate
Coles, 2010 [61]	Yes	Unclear	Unclear	No	No	N/A	Yes	Yes	Yes	4/8	Low
Conrad, 2017 [51]	Yes	No	No	No	Unclear	N/A	Yes	Yes	Yes	4/8	Low
Feir, 2016 [65]	Yes	No	Yes	Yes	No	N/A	Yes	Yes	Yes	6/8	Moderate
Furzer, 2020 [67]	Yes	Unclear	No	Yes	Yes	N/A	Yes	Yes	Yes	6/8	Moderate
Goldstein, 2020 [44]	Yes	Unclear	No	No	Yes	N/A	Yes	Yes	Yes	5/8	Moderate
Hamad, 2019 [62]	Yes	Unclear	Unclear	Yes	Yes	N/A	Yes	Yes	Yes	6/8	Moderate
Hamilton, 2020 [55]	Yes	No	Unclear	Yes	Yes	N/A	Yes	Yes	Yes	7/8	Moderate
Hatzenbuehler, 2017 [56]	Yes	Unclear	Unclear	Yes	No	N/A	Yes	Yes	Yes	5/9	Moderate
Hoynes, 2015 [45]	Yes	Unclear	No	Yes	Yes	N/A	Yes	Yes	Yes	6/8	Moderate
Jia, 2020 [52]	Yes	Unclear	Unclear	Yes	Yes	N/A	Yes	Unclear	Yes	5/8	Moderate
Komro, 2019 [47]	Yes	Yes	Unclear	Yes	Yes	N/A	Yes	Yes	Yes	7/8	High
Kong, 2014 [53]	Yes	No	Unclear	Yes	No	No	Yes	Yes	Yes	5/9	Moderate
Larson, 2019 [66]	Yes	N/A	Yes	No	No	Unclear	Yes	Yes	Yes	4/8	Low
Potochnick, 2017 [57]	Yes	Unclear	Yes	Yes	Yes	N/A	Yes	Yes	Yes	7/8	Moderate
Rosenquist, 2020 [48]	Yes	Unclear	No	No	Yes	N/A	Yes	Yes	Yes	5/9	Moderate
Sudhinaraset, 2020 [63]	Yes	No	No	Yes	No	N/A	Yes	Yes	Yes	5/8	Moderate
Torche, 2019 [58]	Yes	Unclear	Yes	Yes	Yes	N/A	Yes	Yes	Yes	7/8	High
Vargas, 2017 [59]	Yes	Unclear	Unclear	Yes	No	N/A	Yes	Yes	Yes	5/8	Moderate
Venkataramani, 2017 [60]	Yes	No	Unclear	Yes	Yes	N/A	Yes	Yes	Yes	6/8	Moderate

*N/A* not applicable

^a^Only items that received a “Yes” were counted toward the overall score

^b^Ratings were based on the proportion of possible criteria met by the study (≤50% Low; 51–75% Moderate, > 75% High)

**Table 3:** Quality Assessment of Included Randomized Controlled Trial

			JBI Checklist for Randomized Controlled Trials											
	1. True randomization	2. Allocation concealment	3. Similar groups at baseline	4. Participants blinded	5. Those delivering treatment blinded	6. Outcome assessors blinded	7. Identical treatment of groups	8. Follow up complete or differences described	9. Participants analyzed in groups	10. Outcomes measured in same way	11. Reliable outcome measurement	12. Appropriate statistical analysis	Overall score^a^/ Possible score ^a^	Rating^b^
Jagannathan, 2010 [46]	Yes	Yes	Yes	No	No	Unclear	No	Unclear	Yes	Yes	Yes	Yes	7/13	Moderate

*N/A* not applicable

^a^Only items that received a “Yes” were counted toward the overall score

^b^Ratings were based on the proportion of possible criteria met by the study (≤50% Low; 51–75% Moderate, > 75% High)

All quasi-experimental studies were assessed for a temporal relationship between variables, to assess validity of a causal relationship. All were rated as valid since policy implementation clearly preceded the measured outcomes. Most quasi-experimental studies (*n* = 25) [39–45, 47–52, 54–63, 65, 67] performed secondary analyses on existing datasets, so the completeness of follow-up was rated as not applicable, lowering the possible total score for these studies. The single included randomized controlled trial was rated as moderate, with limitations for blinding of participants and outcome assessors, and unclear completeness of follow-up [46].

### Study findings

Findings from included studies are grouped by intervention policy domain with descriptions for how the interventions align with the WHO CSDH framework (Fig. 1). Study findings are reported for each policy domain.

#### Financial policies

Nine quasi-experimental studies [39–45, 47, 48] and the one RCT [46] explored interventions related to financial policy in the USA. Interventions aligned with macroeconomic policies in the WHO CSDH framework [33], such as the Earned Income Tax Credit (EITC) for low-to-moderate-wage earners [39, 41, 42, 45, 47], government expenditure on non-healthcare services [44], and/or minimum wage laws [43, 48]. Other interventions aligned with social protection public policies as a determinant of health, such as Old Age Assistance [40], and the New Jersey Family Development Program [46].

The Earned Income Tax Credit (EITC) reduces personal income tax liability for low-to-moderate workers in the USA, with amounts varying across states and increasing with the number of children in the household [68]. Two studies found no effect of different levels of EITC on adult health behaviours such as smoking and health-related outcomes [39, 41]. Financial stress in lower-income populations has been associated with smoking behaviours [69–71]. Averett and Wang found no statistically significant change in smoking status for Black or Hispanic mothers due to EITC expansion [39], while Braga et al found no statistically significant effect on self-reported overall health, obesity, high blood pressure, functional limitations, emotional problems during adulthood for Black, Hispanic or other racialized adults related to differing levels of EITC received during childhood [41]. The remaining three EITC studies examined intervention effects on birth outcomes with mixed results [42, 45, 47]. Bruckner et al found no significant difference in the odds of very low birthweight between Black and White women receiving the EITC, except in the case of Black women who received the EITC within 2 months prior to delivery, who had increased odds of very low birthweight [42]. Hoynes et al found an association between EITC expansion to a higher maximum available credit amount and reduced incidence of very low birth weight for Black women (0.73%, *p* < 0.01) [45]. Komro et al found that higher levels of EITC were associated with statistically significant improvements in evaluated birth outcomes, such as birth weight and gestational age, for both Black and White women, but not for Hispanic women [47].

One study reported the effects of government expenditure on non-health services on infant mortality [44]. Expenditures included spending on social services, housing, education and environment. Goldstein et al found no statistically significant difference in the association between government spending and infant mortality for infants born to Black, Hispanic, Asian or White mothers [44].

Two studies reported positive effects of higher minimum wage on health outcomes in the USA [43, 48]. Cloud et al evaluated the incidence of HIV cases in Black heterosexual individuals across Metropolitan Statistical Areas with different minimum wage levels. It was found that Metropolitan Statistical Areas with $1.00 higher minimum wage had 27.12% (95% CI 18.06, 35.18) lower incidence of HIV in this population [43]. Rosenquist et al examined infant mortality for Black and White women across States with different minimum wage levels over three decades. They found that the odds of infant mortality decreased for Black women when minimum wage was higher (adjusted OR 0.80, 95% CI 0.68, 0.94), or had seen a larger increase (adjusted OR 0.89, 95% CI 0.82, 0.96) [48].

The USA’s Old Age Assistance (OAA) program expanded in 1935 under the Social Security Act to provide financial benefits to seniors [40]. Balan-Cohen found that mortality due to preventable, behavioural or cardiovascular causes was reduced for Black (12%) and White (17%) OAA recipients in non-Southern states, but the statistical difference between groups was not evaluated [40].

Jagannathan et al found mixed results in an RCT to examine the mental health effects of the New Jersey Family Development Program (FDP) welfare reform, which imposed stricter rules for low-income mothers by denying additional cash benefits to mothers of children already receiving benefits, adding work/training requirements and rapid withholding of benefits for program non-compliance [46]. It was found that Black women subject to FDP reform had decreased incidence of clinically diagnosed anxiety disorders (−15.3%, *p* < 0.05) and clinically diagnosed depressive disorders (−2.1%, *p* < 0.05) compared to Black women who were not subject to the reform [46]. Hispanic women subject to FDP reform had increased incidence of clinically diagnosed depressive disorders (68%, *p* < 0.05) compared to Hispanic women who were not subject to the reform [46].

#### Nutrition safeguards

Five studies evaluated interventions to improve access to nutritious food in the USA. Within the WHO CSDH framework [33], these public policies impact socioeconomic position in terms of income and gender, to affect material circumstances in terms of food security (Fig. 1). Two studies evaluated the Special Supplemental Nutrition Program (SNAP) [50, 51], two studies evaluated the Special Supplemental Nutrition Program for Women, Infants, and Children (WIC) [49, 53], and one study evaluated the National School Lunch Program (NSLP) [52].

SNAP, the USA’s largest federal nutritional assistance program that provides food purchasing cards for eligible low-income individuals and families [72], was evaluated by two studies that found mixed results. Conrad et al compared age-adjusted mortality for SNAP participants to eligible non-participants of the same race or ethnicity and found a significantly higher risk of mortality from all causes in Black, Hispanic and White SNAP participants and higher risk of mortality due to diabetes for Black SNAP participants compared to eligible non-participants [51]. Authors suggest that participants face greater hardships than eligible non-participants and are therefore less likely to access medical care [51]. Booshehri et al evaluated SNAP by comparing prevalence of diet-related morbidities, such as cardiovascular conditions, before and after changes to SNAP enrollment requirements at age 60 [50]. The analysis applies specifically to individuals aged 56–64 who itemized deductions on their tax return and met SNAP eligibility upon reaching age 60 [50]. The reduction in prevalence of hypertension between ages 56–59 and 60–64 was greater for Black populations than Hispanic or White, and the reduction in prevalence of angina and stroke was greater for Hispanic populations than Black or White [50].

Two studies evaluated Special Supplemental Nutrition Program for Women, Infants, and Children (WIC) benefits [49, 53], which provide federal grants to states to provide food, health care referrals and nutrition education to at-risk pregnant women and children up to age 5 [73]. Arons et al found no significant difference in measures of socioemotional development for Black children who received WIC, compared to children of all races who receive WIC [49]. Kong et al investigated diet quality of mothers and children following changes to WIC to provide more whole grains, fruits, vegetables and fewer saturated fats and found no significant change in diet quality for mothers [53]. Black children had an increase in consumption of sugar-sweetened beverages and Hispanic children had improved diet quality and reduced saturated fat intake [53].

Jia et al evaluated diet quality in children following changes to the USA’s National School Lunch Program to increase the amount and variety of fruits and vegetables offered, restrict grains to whole grains and restrict sugar-sweetened beverages to non-fat milk only [52]. It was found that Black students increased their overall fruit and vegetable intake, while Hispanic students reduced their weekday fruit and vegetable intake [52].

#### Immigration

Eight studies evaluated the effect of immigration-related policies on Hispanic/Latinx populations in the USA (Table 1). Six studies evaluated the effects of anti-immigration on health or other health outcomes [54, 56–59], and two studies evaluated the effect of the Deferred Action for Childhood Arrivals (DACA) policy [55, 60]. In alignment with the WHO CSDH framework [33], each of these policies affect governance and influence one’s socioeconomic position based on race and/or ethnicity (Fig. 1).

The six studies that examined anti-immigration policies found that anti-immigrant social climates and more aggressive immigration law enforcement negatively affected Hispanic and Latinx populations [54, 56–59]. Of the studies evaluating the effect of anti-immigration policies on mental and physical health outcomes for Hispanic and Latinx populations, Bruzelius et al did not find a significant change in Latinx mental health after enactment of national policies that led to increased immigration arrests [54]. The study by Hatzenbuehler et al found that Latinx people in states with more exclusionary immigration policies reported more frequent poor mental health days [54, 56]. In a survey of Latinx adults across different American states, Vargas et al found a relationship between punitive anti-immigration policies and a decreased likelihood of reporting overall health as optimal [59]. Torche and Sirois specifically examined birthweight of infants born to immigrant Latina mothers before and after Arizona’s Senate Bill SB1070, which increased immigration policy enforcement [58]. A statistically significant decline in birthweight was found for infants born in the latter half of 2010, whose mothers were exposed to the passage of the law during their pregnancies [58]. Potochnik et al evaluated the effect of Federal 287(g) program, which enabled increasingly aggressive immigration law enforcement, and found increased food insecurity for Mexican non-citizen households with children [57].

Two studies evaluated the health effects of DACA [55, 60], a program that protected young immigrants who were brought to the USA as children from deportation [74]. Hamilton et al found significant improvements in birth outcomes for infants born to DACA-eligible mothers [55]. Venkataramani et al found that implementation of DACA was associated with significant reductions in psychological distress for those who were eligible for the program [60].

#### Family and reproductive policies

Three studies examined the effects of policies for reproductive rights and paid family leave in the USA using quasi-experimental designs [61–63]. These health and social protection policies align with the structural level of the WHO CSDH framework [33], affecting socioeconomic position in terms of gender and income (Fig. 1).

Coles et al and Sudhinaraset et al found negative health effects of restrictive abortion policies by comparing health outcomes in states with more restrictive or less restrictive abortion policies [61, 63]. In states with restricted Medicaid funding for abortions, Black minors had higher rates of unplanned births than in states without such restrictions, while there was no statistically significant difference for White or Hispanic minors [61]. In less restrictive states, Black women had a lower risk of low birth weight than in more restrictive states [63].

In evaluating the effect of parental leave policy, Hamad et al found that a 6-week paid parental leave was not sufficient to improve breastfeeding outcomes for Black mothers, who were less likely than White mothers to report breastfeeding at 12-months post-partum, while Hispanic mothers who received 6-week paid parental leave were more likely to report exclusive breastfeeding at 6-months postpartum [62].

#### Policies for indigenous populations

Three studies examined policies designed for Indigenous populations [64–66]. Two studies evaluated policies designed to improve living conditions for Indigenous populations in Australia through Indigenous Land and Sea Management Programs (ILSMPs) and Alcohol Management Programs (AMPs) [64, 66], while one study evaluated the generational effect of Canada’s residential school system [65]. The ILSMPs and AMPs align with governance, social policies and cultural values as structural determinants in the WHO CSDH framework [33], impacting socioeconomic position in terms of race, to affect material and health outcomes (Fig. 1). Relevant health outcomes for Indigenous populations are often more holistic and tied to the land on which they reside [75, 76]. The forced relocation and cultural erasure as a result of Canada’s residential school system affected multiple structural determinants based on race, with profound negative effects on material circumstances, behaviours, biological and psychosocial outcomes [77] [cite].

In Australia, AMPs have been used by governments since a 2001 inquiry into domestic violence, injury and deaths that found that historical and ongoing colonialism created conditions that put Indigenous communities at higher risk for alcohol-related harms. Under the AMP, alcohol availability is highly regulated and illicit possession or consumption were strictly penalized [64]. The effects of AMPs were mixed, and while community members reported less violence and increased feelings of safety, they also reported that there was more substance use and law enforcement [64]. Also in Australia, the federal government implemented ILSMPs, which seek to encourage Indigenous land management through creating employment and economic opportunities in land and sea management activities [66]. Implementation of ILSMPs had several positive effects on community members, with the majority reporting satisfaction for the health of the land, their legal right to the land and business ownership, and improvements to information and communications technology access [66].

Feir investigated traditional Western health outcomes for First Nations, Métis and Inuit children whose mothers attended residential schools, and found they had a higher average BMI than for First Nations, Métis and Inuit children whose mothers did not attend residential schools [65]. Educational outcomes were also reported, including increased suspensions, expulsions and worse school experiences for children whose mothers attended residential schools [65].

#### Environmental

One study evaluated the effects of an environmental policy on health outcome inequities [67]. Furzer and Miloucheva analyzed the effect of Clean Air Act regulations in the USA [67]. In alignment with the WHO CSDH framework [33], the study describes how the Clean Air Act affects public policies in terms of race to impact biological outcomes (Fig. 1). Furzer and Miloucheva found that air pollution limits were less likely to be attained in counties with higher proportions of Black or racialized residents, resulting in 6.8–18% more COVID-19 deaths in these populations than in regions that maintained air pollution limits [67].

## Discussion

This systematic review explores evidence for structural-level interventions that affect racial health inequities through determinants outlined by the WHO CSDH framework [33] (Fig. 1). Among the 29included studies, only 18 interventions specifically address race- or ethnicity-based inequities; 13 of which act through improving socioeconomic circumstances. Most interventions were targeted toward low-income populations, except for immigration policies and policies for Indigenous populations. There were no studies of policies designed to mitigate anti-Black racism, a notable gap that suggests anti-Blackness within governments and academia.

Overall, studies reported mixed effects of interventions that affect the structural determinants of health, as defined by the WHO CSDH framework. Studies of financial and nutrition safeguard policies largely found no or mixed effects, with the notable exception of two moderate-quality studies of minimum wage policies which were shown to reduce HIV incidence and improve birth outcomes for Black populations. Discriminatory policies, such as anti-immigration enforcement and abortion restrictions were shown to negatively impact birth outcomes and mental health outcomes for affected Hispanic and Black populations. Conversely, two moderate-quality studies of DACA found improvements to birth and mental health outcomes for DACA-eligible populations. Findings from policies for Indigenous populations were mixed but provided some evidence that enhancing self-governance may lead to improved outcomes. While all included interventions had potential to affect structural determinants of health according to the WHO CSDH framework (Fig. 1), findings were inconsistent for different racial and ethnic populations. Findings from this review support Critical Race Theory’s tenet that racism functions differently for different races according to a racial hierarchy [1], and support the need for population-specific interventions rather than broader, non-targeted interventions. Policies that address systemic barriers encountered by different racialized groups may be more effective than those applied to broader populations, as suggested by Assari, 2018, who proposes that the intersection of race, socioeconomic status and gender shape exposures to risk and protective factors [78]. For example, findings of a policy analysis by Carvalho et al, 2021, support a targeted approach, reporting positive impacts on health equity by policies directly addressing Black maternal health [79]. The studies included in this review that targeted specific populations were also more likely to show positive effects on inequities, for example DACA for Hispanic populations in the USA [55, 60] and the Indigenous Land and Sea Management Programs in Australia [66]. Based on the results of this systematic review, policies designed to address racial health inequities experienced by specific populations are more likely to reduce health disparities than broad policies that target populations based solely on socioeconomic status.

This review illustrates a scarcity of evidence evaluating the health impact of interventions addressing structural racism [4, 22]. In a recent systematic review of studies of institutionalized racism in the top 50 highest-impact journals in the USA between 2002 and 2015, the term was included in the article abstract or title of only 25 papers and as a critical concept in only 16 papers [23]. Some limitations of the body of evidence included in this review may be due to limited data available regarding race. Many included studies were conducted in the USA, where racial and ethnic data in public systems has limited consistency, reliability and comprehensiveness across states [80–83]. These data challenges have been reported in the Canadian context as well, where race-based data are either not collected, reported or account for small proportions of the dataset, raising issues of privacy and limiting analyses of intersectional identities such as ethnicity, and immigration status [84–86]. Some critical race scholars have argued that the lack of data reflects an ongoing denial of the salience of racism as a determinant of health in the Canadian context.

A single RCT was included in this systematic review [46]. This trial examined the effect of the New Jersey FDP Welfare Reform’s stricter rules on the mental health of low-income mothers receiving benefits. While the study found that Black women subject to FDP Reform had a lower incidence of clinically diagnosed anxiety or depressive disorders, the study’s findings are limited by its design. Study authors chose clinical diagnosis of a mental health disorder as their primary outcome, rather than self-reported symptoms, citing discrepancies between female self reports of mental distress and clinician reports [87]. The biases faced by women seeking health care are well-documented [88–91] and for Black women in particular [92–95]. Authors also cited a study that demonstrated depressive symptoms are often short-lived, but this study reported on depressive symptoms due to bereavement, which may not be transferable to the experiences of low-income mothers seeking welfare [96]. Authors did not address that Black women face barriers to accessing mental healthcare [97–99]. Authors instead discuss so-called “welfare heritage” of Black women with more frequent and longer use of welfare services [46]. The methodological biases and racial bias in the design of this RCT highlight the need for racial equity-centered evaluations of interventions.

Notably, the interventions examined by the studies included in this review were largely limited to one policy domain, for example, interventions that impacted finances or interventions that affected immigration rights. However, calls to action to counter structural racism have emphasized the need for broad, all-policy approaches [100–103]. It is perhaps not unexpected that individual studies of single-policy interventions found mostly mixed or no effect on health inequities, as a systemic issue requires systemic solutions. Two studies of minimum-wage levels and one study of EITC expansion [43, 45, 48] found positive effects of increased minimum wage on health inequities, supporting unconditional cash transfers that can impact daily living. Similarly, two studies of DACA found reduced inequities [55, 60], possibly because the opportunities granted by DACA affect many areas of life.

A limitation of this systematic review is that for most included studies it is difficult to attribute change in outcomes to the intervention, which is a consistent challenge reported previously in policy evaluations [104–106]. The most rigorous evidence for the effect of an intervention would be produced by a randomized controlled trial design where study groups receive identical treatment except for the intervention under study. Due to ethical and logistical barriers however, randomized controlled trials are rarely feasible, which is illustrated in this review with nearly all included studies using a quasi-experimental design. Given the pervasive effects of structural racism, for studies that conducted comparisons between racialized groups, it is unlikely that the groups had similar baseline experiences apart from the intervention [100, 107–109]. This limits both the effect attributable to the intervention, as well as the magnitude of the effect as the intervention is but one of many differences between groups. For these reasons, changes in outcome for the same population group prior to and after implementation of an intervention will provide more rigorous evidence for the effect of an intervention.

This review had several limitations in its design. Study selection was guided by the WHO CSDH framework (Fig. 1), to ensure that included interventions target the structural determinants of health. This review found that interventions must be tailored for specific racialized groups, but the guiding WHO framework was applied to all populations and may not truly reflect the determinants of health for specific groups. Studies were also limited to those conducted in OECD countries, to reflect Canada’s context of democratic government and high-income economy most closely. This limits the findings of this review to other high-income democracies, and results may not necessarily apply to low- or middle-income countries. Structural racism continues to impact health inequities in low- and middle-income countries, and further research is required to investigate potential interventions to mitigate these inequities [110–112].

The findings of this systematic review contribute to an understanding of how to make meaningful change to improve racial health inequities. Findings are complementary to those of other policy interventions that have not measured health outcomes, but demonstrate impact on the social determinants of health [100]. For example, the Purpose Built Communities in Atlanta, USA, which engaged community residents in designing and implementing neighbourhood design and education programs, has shown positive effects for crime, housing, and employment [4]. This intervention was effective likely because it was multilevel and upstream, targeting living conditions to improve health outcomes [113]. The interventions in this systematic review were largely limited to one policy domain and while they had the potential for upstream impact on health outcomes (in alignment with the WHO CSDH framework, Fig. 1), most were insufficient in scope to cause measurable improvements. Structural racism functions across multiple domains which are mutually reinforcing. While policy action in one domain may reduce racial health inequities, this may not be sufficient to counter the impacts of racism in other domains. For policy interventions to effect measurable and meaningful improvements to racial health inequities, policies must be designed to synergize across domains for upstream impact on daily living conditions [2, 4, 14, 113].

### Implications

There are several implications for the reduction of racial health inequities from the findings of this systematic review.

1. Structural racism must be addressed through comprehensive, upstream policy interventions that improve daily living conditions. Policies that affect the structural determinants of health, in alignment with the WHO’s CSDH framework, toimprove socioeconomic status and opportunities, e.g., minimum wage increases and DACA, show promise. Policies specifically designed for racialized populations may be more effective in reducing disparities than policies targeting populations based solely on socioeconomic status.
2. Discriminatory policies, such as restrictions to abortion access or anti-immigration policies, have demonstrable harms to racialized populations.
3. Research on the effects of interventions for structural health inequities is lacking, particularly for Black populations. Since research is conducted and funded by structures that perpetuate racism, such as academia and government, significant efforts must be made to ensure the focus of interventions and research is equitable.
4. Research on interventions to mitigate structural racism would benefit from well-designed studies on policies targeting multiple domains, such as income, employment, education and built environment. Research that uses multiple methods and is co-designed with those who have lived experience of structural racism is required. While randomization may not always be possible, studies should strive to include intervention and control groups of the similar racialized identity, multiple measurements over sufficient time to see the effect of an intervention and validated relevant measures of outcomes.

## Conclusions

Structural racism remains a pervasive issue with inequitable effects on health for racialized populations. One-dimensional policy interventions lack the impact on daily living conditions to effect measurable change in health outcomes. The WHO CSDH framework defines pathways through which interventions can address the systemic barriers faced by racialized groups and impact the structural determinants of health. Future versions of this framework should consider specific contexts for different racialized populations, to ensure that it can be applied broadly as intended. In the current state of research, few interventions that target the structural determinants of health have been evaluated for their effect on health outcome inequities. Significant and long-term investments into dedicated programs for research, specific and unique to the needs of specific racialized groups, are necessary to address the root causes of structural inequities. While the overall goal of improving health outcomes is common for all populations, concerted efforts to develop, implement and evaluate policies that address the unique contexts of structural racism facing different racialized populations are required to reduce inequities.

## Supplementary Information

**Additional file 1.** Search Strategies.

**Additional file 2.** Excluded Studies.

## Data Availability

The datasets used and/or analysed during the current study are available from the corresponding author on reasonable request.
