# Supplementary material for: Structural interventions that affect racial inequities and their impact on population health outcomes: a systematic review
Source: BMC Public Health. 2022 Nov 24;22:2162. doi: 10.1186/s12889-022-14603-w (PMC9685079; doi:10.1186/s12889-022-14603-w)
Supplement: Supplementary file 1 — Additional file 1. Search Strategies. [file 12889_2022_14603_MOESM1_ESM.docx]

Additional file 1. Search Strategies

The initial search strategies were designed for EBSCO Medline, then adapted to other sources, such as Google Scholar. Medline was searched for publications between January 2005 and October 2022. Four independent searches were run, and all identified studies were deduplicated and assessed for eligibility.

Geographic limiters provided by the research group were as follow:

| **G20 Countries:** <https://www.dfat.gov.au/trade/organisations/g20/Pages/g20>  Plain language syntax:  AB (Argentin* OR Australia* OR Brazil* OR Canad* OR China OR Chinese OR France OR French OR German* OR India OR Indian OR Indonesia* OR Italian OR Italy OR Japan* OR “South Korea” OR Korean OR Mexic* OR Russia* OR “Saudi Arabia*” OR “South Africa*” OR Turkey OR Turkish OR “United Kingdom” OR “United States” OR America* OR “European Union”  **United Kingdom** is comprised of the following individual countries = AB (England OR “Northern Ireland” OR Wales OR Welsh OR Scotland OR Scottish)  **EU member countries**: <https://europa.eu/european-union/about-eu/countries_en>  AB (Austri* OR Belgium OR Belgian OR Bulgaria* OR Croatia* OR Cyprus OR Czech* OR Denmark OR Danish OR Estonia* OR Finland OR Finnish OR France OR French OR German* OR Greece OR Greek OR Hungar* OR Ireland OR Irish OR Italy OR Italian OR Latvia* OR Lithuania* OR Luxembourg OR Malta OR Netherlands OR Holland OR Poland OR Polish OR Portugal OR Portuguese OR Romania* OR Slovakia* OR Slovenia* OR Spain OR Sweden)  **Add because they are not included in the G20 countries list or the EU, but are similar to other high-income countries listed in the G20 and EU sets:**  “New Zealand”  Norway OR Norwegian  **Terms translated for EBSCO Medline (all countries are alphabetical from the combined lists above)**  (MH "Austria") OR (MH "Argentina") OR (MH "Australia+") OR (MH "Belgium") OR (MH "Brazil") OR (MH "Bulgaria") OR (MH "Canada+") OR (MH "China+") OR (MH "Czech Republic") OR (MH "Croatia") OR (MH "Cyprus") OR (MH "Denmark+") OR (MH "European Union") OR (MH "Europe") OR (MH "England+") OR (MH "Estonia") OR (MH "Finland") OR (MH "France+") OR (MH "Greece") OR (MH "Germany") OR (MH "Hong Kong") OR (MH "Hungary") OR (MH "Italy+") OR (MH "Ireland") OR (MH "India") OR (MH "Indonesia") OR (MH "Japan+") OR (MH "Korea") OR (MH "Republic of Korea") OR (MH "Latvia") OR (MH "Lithuania") OR (MH "Malta") OR (MH "Netherlands") OR (MH "Norway+") OR (MH "New Zealand") OR (MH "Northern Ireland") OR (MH "North America") OR (MH "Portugal") OR (MH "Poland") OR (MH "Romania") OR (MH "Slovenia") OR (MH "Slovakia") OR (MH "Saudi Arabia") OR (MH "Scotland") OR (MH "Switzerland") OR (MH "Spain") OR (MH "Scandinavian and Nordic Countries") OR (MH "Sweden") OR (MH "Wales") OR (MH "Turkey") OR (MH "South Africa") OR (MH "Russia") OR (MH "United Kingdom") OR (MH "United States+") OR (MH "Wales") |
| --- |

**Search 1 – Focused on social supports, such as (un)employment benefits, government-provided pensions, government-provided health insurance or universal health care, social assistance and/or social welfare programs such as disability pension programs.**

S1: (MH "Government") OR (MH "Local Government") OR (MH "State Government") OR (MH "Government Agencies") OR (MH "Federal Government") OR (MH "Government Programs") OR (MH "Government Regulation") OR (MH "Public Policy") OR (MH "Legislation as Topic") OR (MH "Social Control, Formal") OR (MH "Policy Making") OR (MH "Mandatory Programs+") OR (MH "Fiscal Policy") OR (MH “Taxes”) OR (MH "Financing, Government") OR (MH "Antitrust Laws") OR (MH "Privatization")

S2: AB ((govern* OR politic*) N3 (local OR municipal* OR state OR provinc* OR federal OR program* OR policy OR policies OR legislat* OR law OR will OR decision*))

S3: ( (MH "Political Systems") OR (MH "Socialism") OR (MH "Democracy") ) OR ( AB (Decoloni* OR Indigeni* OR negotiat* OR collaborat* OR compromis* OR "shared-decision*") )

S4: ( AB ((Determinants OR disparit* OR inequit* OR bias* OR marginali*) N3 (Structural OR Institutional)) ) OR ( TI (determinants OR disparit* OR equit* OR equalit* OR inequit* OR inequal* OR unequal* OR disadvantage* OR upstream OR barriers OR facilitators) )

S5: (MH "Social Determinants of Health") OR (MH "Health Equity") OR (MH "Health Status Disparities") OR (MH "Healthcare Disparities") OR (MH "Health Status") OR (MH "Socioeconomic Factors") OR (MH "Social Conditions") OR (MH "Quality of Life") OR (MH "Right to Health") OR (MH "Human Rights+") OR (MH "Civil Rights") OR (MH "Women's Rights") OR (MH "Social Responsibility") OR (MH "Social Justice") OR (MH “Social Marginalization”) OR (MH "Civil Rights")

S6: (MH "Public Assistance") OR (MH "Employee Retirement Income Security Act") OR (MH "Old Age Assistance") OR (MH "Social Security") OR (MH "Retirement") OR (MH "Pensions") OR (MH "Workers' Compensation")

S7: AB (pension OR retire* OR social OR security OR disability OR universal OR basic OR unemployment OR child OR parent* OR mother) N3 (income OR supplement OR payment OR assistance OR compensation OR benefit OR welfare OR support OR subsidi* OR stipend)

S8: (MH "Single-Payer System") OR (MH "Medical Assistance") OR (MH "Universal Health Insurance") OR (MH "National Health Insurance, United States") OR (MH "Universal Health Care") OR (MH "Medicare+") OR (MH "Medicaid") OR (AB "single payer" OR "tax funded" OR "universal health care" OR "universal health insurance")

S9: ( TI ((public OR universal OR national OR provincial OR territorial) N2 (health OR dental OR mental OR insurance)) )

S10: AB ((public OR universal OR national OR provincial OR territorial) N2 (health OR dental OR mental OR insurance))

S11: (MH "Insurance Coverage") OR (MH "Aid to Families with Dependent Children") OR (MH "Insurance") OR (MH "Insurance, Health") OR (MH "Insurance Benefits") OR (MH "Insurance, Life") OR (MH "Patient Protection and Affordable Care Act") OR (MH "Not-For-Profit Insurance Plans") OR (MH "Medical Savings Accounts") OR (MH "Insurance, Vision") OR (MH "Insurance, Surgical") OR (MH "Insurance, Psychiatric") OR (MH "Insurance, Pharmaceutical Services+") OR (MH "Insurance, Long-Term Care") OR (MH "Insurance, Major Medical") OR (MH "Insurance, Hospitalization") OR (MH "Insurance, Health, Reimbursement") OR (MH "Insurance, Dental") OR (MH "Insurance, Accident") OR (MH "Health Insurance Portability and Accountability Act") OR (MH "Health Benefit Plans, Employee") OR (MH "Community-Based Health Insurance") OR (MH "Children's Health Insurance Program") OR (MH "Insurance, Physician Services+") OR (MH "Insurance, Nursing Services") OR (MH "Insurance, Medigap") OR (MH "Insurance, Disability") OR (MH "Deductibles and Coinsurance")

S12: (MH "Implementation Science") OR (MH "Health Plan Implementation") OR (MH "Regional Health Planning") OR (MH "Social Planning") OR (MH "Diffusion of Innovation") OR (AB "knowledge translation" OR "diffusion of innovation" OR implement* OR initiat* OR "roll-out" OR intervention)

S13: TI (implementation AND (govern* OR policy OR policies OR initiative))

S14: (S1 OR S2 OR S3) AND (S4 OR S5) AND (S6 OR S7)

S15: (S1 OR S2 OR S3) AND (S4 OR S5) AND (S8 OR S9 OR S10 OR S11)

S16: (S1 OR S2 OR S3) AND (S4 OR S5) AND (S8 OR S9 OR S10 OR S11) AND S12

S17: AB (Argentin* OR Australia* OR Austri* OR Belgium OR Belgian OR Brazil OR Bulgaria* OR Canad* OR China OR Chinese OR Croatia* OR Cyprus OR Czech* OR Denmark OR Danish OR Estonia* OR Finland OR Finnish OR France OR French OR German* OR Greece OR Greek OR Hungar* OR Ireland OR Irish OR Italy OR Italian OR India OR Indian OR Indonesia* OR Latvia* OR Lithuania* OR Luxembourg OR Malta OR Mexic* OR Netherlands OR Holland OR Poland OR Polish OR Portugal OR Portuguese OR Romania* OR Slovakia* OR Slovenia* OR Spain OR Sweden OR England OR “Northern Ireland” OR Wales OR Welsh OR Scotland OR Scottish OR Japan* OR “South Korea” OR Korean OR Russia* OR “Saudi Arabia*” OR “South Africa*” OR Turkey OR Turkish OR “United Kingdom” OR “United States” OR America* OR “European Union” OR "New Zealand" OR Norway OR Norwegian) OR (MH "Austria") OR (MH "Argentina") OR (MH "Australia+") OR (MH "Belgium") OR (MH "Brazil") OR (MH "Bulgaria") OR (MH "Canada+") OR (MH "China+") OR (MH "Czech Republic") OR (MH "Croatia") OR (MH "Cyprus") OR (MH "Denmark+") OR (MH "European Union") OR (MH "Europe") OR (MH "England+") OR (MH "Estonia") OR (MH "Finland") OR (MH "France+") OR (MH "Greece") OR (MH "Germany") OR (MH "Hong Kong") OR (MH "Hungary") OR (MH "Italy+") OR (MH "Ireland") OR (MH "India") OR (MH "Indonesia") OR (MH "Japan+") OR (MH "Korea") OR (MH "Republic of Korea") OR (MH "Latvia") OR (MH "Lithuania") OR (MH "Malta") OR (MH "Netherlands") OR (MH "Norway+") OR (MH "New Zealand") OR (MH "Northern Ireland") OR (MH "North America") OR (MH "Portugal") OR (MH "Poland") OR (MH "Romania") OR (MH "Slovenia") OR (MH "Slovakia") OR (MH "Saudi Arabia") OR (MH "Scotland") OR (MH "Switzerland") OR (MH "Spain") OR (MH "Scandinavian and Nordic Countries") OR (MH "Sweden") OR (MH "Wales") OR (MH "Turkey") OR (MH "South Africa") OR (MH "Russia") OR (MH "United Kingdom") OR (MH "United States+") OR (MH "Wales")

S18: S13 OR S14 OR S15 OR S16

S19: S17 AND S18

S20: (MH "Vulnerable Populations")

S21: (MH "Prisoners") OR (MH "Enslaved Persons") OR (MH “Juvenile Delinquency”) OR (AB (Inmates OR Offender OR Incarcerated OR Incarceration OR Prisoner OR detainee))

S22: (MH "Homeless Persons") OR (MH “Homeless Youth”) OR (MH "Transients and Migrants") OR (AB (homeless* OR transient* OR unhoused) OR (AB (encampment OR shelter OR (“precariously housed” OR “precarious housing”) OR AB ((precarious* OR temporary) N3 (hous* OR residen* OR shelter*)))

S23: ( (MH "Transgender Persons") OR (MH "Sexual and Gender Minorities") OR (MH "Intersex Persons") OR (MH “Gender Identity”) OR (MH "Homosexuality+") OR (MH "Transsexualism") OR (MH "Bisexuality") ) OR ( AB (Transgender OR Trans-masculine OR transmasculine OR Transfeminine OR Trans-feminine OR Non-binary OR “Non-Conform*” OR nonconform* OR “Gender Fluid*” OR “Gender diverse” OR “Gender expansive” OR Genderqueer OR Gay OR Lesbian OR Bisexual OR Intersex* OR Pansexual OR Asexual* OR Queer OR “Two-Spirit” OR “Sexual Minority” OR “LGBT*” OR “GLBT*” OR questioning) )

S24: (MH "Indigenous Peoples") OR (MH "American Native Continental Ancestry Group") OR (MH "Oceanic Ancestry Group") OR (MH "Indians, North American") OR (MH "Indians, South American") OR (MH "Indians, Central American") OR (MH "Inuits") OR (MH "Alaska Natives") OR AB (Indigenous OR Aboriginal OR Maori OR “Torres Strait Islander” OR “First Nation” OR Metis OR Inuit OR Inuk OR Innu OR “American Indian” OR “Alaska Native” OR “Native Hawaiian” OR “Pacific Islander” OR Sami OR “Two Spirit”)

S25: AB (Immigrant* OR Refugee OR Undocumented OR Migrant* OR Asylum OR “Asylum Seek*”) OR (MH "Emigrants and Immigrants")

S26: (MH "Jews") OR (MH “Islam”) OR ( AB (Jew* OR Judaism OR Islam* OR Muslim OR Hindu* OR Sikh* OR Roma OR "religious minorit*")

S27: (MH "African Americans") OR (MH "African Continental Ancestry Group") OR AB (Black OR "African-American" OR African OR Caribbean OR Afro* OR "BAME" OR “Black, Asian and minority ethnicity”) OR AB ("Mixed-race" OR biracial OR "bi-racial" OR "multiracial" OR "multi-racial"OR raciali*)

S28: AB (Deaf* OR Blind* OR (visual* N3 impair*)) OR ( AB (Wheelchair OR “mobility device” OR walker) N3 use*) ) OR (MH "Disabled Persons") OR (MH "Disabled Children") OR (MH "Mentally Disabled Persons") OR (MH "Persons With Hearing Impairments") OR (MH "Visually Impaired Persons") OR (MH "Amputees") OR (MH "Mentally Ill Persons") OR (MH "Homebound Persons") OR (MH "Bedridden Persons") OR (MH "Vision Disorders+") OR (MH "Blindness+") OR (MH "Deaf-Blind Disorders+") OR (MH "Hearing Loss+") OR (MH "Deafness") OR (MH "Vision, Low") OR (MH "Self-Help Devices") OR (MH "Wheelchairs") OR (MH "Communication Aids for Disabled") OR (MH "Hearing Aids") OR (MH "Cochlear Implants") OR (MH "Auditory Brain Stem Implants") OR (MH "Correction of Hearing Impairment") OR (MH "Hearing Disorders") OR AB (Deafness OR Blindness OR Disabled OR Disability)

S29: ( (MH "Working Poor") OR (MH "Student Dropouts") OR (MH "Single Person") OR (MH "Women, Working") OR (MH "Students") ) OR ( (AB (Worker* OR staff OR Laborer* OR labourer* OR employee*) ) OR ( AB (Unstabl* OR marginal* OR precarious OR temporary OR migrant OR undocument* OR immigrant OR informal OR non-standard) N3 (work* OR labour* OR labor* OR job OR staff OR employee*) ) )

S30: (MH "Pregnant Women") OR (MH "Parents") OR (MH "Single Parent") OR (MH "Mothers") OR (MH "Fathers") OR (MH "Legal Guardians") OR (MH "Grandparents") OR (MH "Caregivers") OR AB (parent* OR caregiver* OR guardian OR mother OR father OR grandparent OR family)

S31: (MH "Hispanic Americans") OR (MH "Mexican Americans") OR AB (Latina OR Latino OR Latinx OR Hispanic OR “Spanish-speaking”)

S32: (MH "Drug Users") OR (MH "Alcoholics") OR AB ("people who use drugs" OR "people who inject drugs" OR "persons who use drugs" OR "persons who inject drugs" OR "PWID" OR "PWUD")

S33: (MH "Prisoners") OR (MH "Enslaved Persons") OR (MH “Juvenile Delinquency”) OR (AB (Inmates OR Offender OR Incarcerated OR Incarceration OR Prisoner OR detainee))

S34: (MH "Arabs") OR (AB (Arab* OR “Middle East*” OR “North African” OR “Arabic-speaking” OR "Farci-speaking")

S35: (MH "Asian Americans") OR (MH "Asian Continental Ancestry Group") OR AB (Asian OR “Chinese-speaking” OR “Cantonese-speaking” OR “Mandarin-speaking”)

S36: (MH "Ethnic Groups") OR AB ( (Ethnic* OR ancestry) )

S37: (MH "Aged") OR (MH "Frail Elderly") OR (MH "Aged, 80 and over") OR (AB (Elder* OR senior* OR “older adult” OR frail* OR aging OR ageing)

S38: MH "Child, Foster") OR (MH "Child, Adopted") OR (MH "Child, Abandoned") OR (AB (“foster child*” OR “pre-adopted”))

S39: (MH "Adolescent") OR (MH "Young Adult") OR (MH "Minors") OR (AB (child* OR adolescen* OR teen* OR "emerging adult" OR "young adult"))

S40: (MH "Child") OR (MH "Child, Preschool") OR (AB (child* OR toddler OR pre-school* OR preschool*)

S41: (MH "Infant") OR (MH "Infant, Newborn") OR (MH "Infant, Premature") OR (MH "Infant, Extremely Premature") OR (MH "Infant, Low Birth Weight") OR (MH "Infant, Very Low Birth Weight") OR (MH "Infant, Extremely Low Birth Weight") OR (MH "Infant, Small for Gestational Age") OR (AB (Infant OR baby OR babies OR toddler* OR preemie OR premature))

S42: (S20 OR S21 OR S22 OR S23 OR S24 OR S25 OR S26 OR S27 OR S28 OR S29 OR S30 OR S31 OR S32 OR S33 OR S34 OR S35 OR S36) AND (S37 OR S38 OR S39 OR S40 OR S41)

S43: (S1 OR S2 OR S3) AND (S6 OR S7 OR S8 OR S9 OR S10 OR S11) AND (S20 OR S21 OR S22 OR S23 OR S24 OR S25 OR S26 OR S27 OR S28 OR S29 OR S30 OR S31 OR S32 OR S33 OR S34 OR S35 OR S36) AND S17

S44: ((S4 OR S5) AND (S6 OR S7 OR S8 OR S9 OR S10) AND (S20 OR S21 OR S22 OR S23 OR S24 OR S25 OR S26 OR S27 OR S28 OR S29 OR S30 OR S31 OR S32 OR S33 OR S34 OR S35 OR S36) AND (12 OR 13)) AND S17

S45: (S4 OR S5) AND S11 AND (S20 OR S21 OR S22 OR S23 OR S24 OR S25 OR S26 OR S27 OR S28 OR S29 OR S30 OR S31 OR S32 OR S33 OR S34 OR S35 OR S36) AND S17

S46: S43 OR S44 OR S45

S47: S43 OR S44 OR S45

S48: S46 not S47

**Search 2 – Focused on the intersection between policing, justice, racism or discrimination and racialized populations**

S1: (MH "Ethnic Groups") OR AB (Ethnic*)

S2: (MH "Asian Americans") OR (MH "Asian Continental Ancestry Group") OR AB (“East Asian” OR “South Asian” OR Asian)

S3: (MH "Arabs") OR (AB (“Middle East*” OR Arabic OR Arab OR Palestin*)

S4: (MH "Hispanic Americans") OR (MH "Mexican Americans") OR AB (Latina OR Latino OR Latinx OR Hispanic OR Chicano OR Chicana)

S5: AB ("Mixed-race" OR biracial OR "bi-racial" OR "multiracial" OR "multi-racial"OR racializ* OR racialis*)

S6: (MH "African Americans") OR (MH "African Continental Ancestry Group") OR AB (Black OR "African-American" OR Diaspor* OR African OR Caribbean OR Afro* OR "BAME" OR “Black, Asian and minority ethnicity”)

S7: (MH "Indigenous Peoples") OR (MH "American Native Continental Ancestry Group") OR (MH "Oceanic Ancestry Group") OR (MH "Indians, North American") OR (MH "Indians, South American") OR (MH "Indians, Central American") OR (MH "Inuits") OR (MH "Alaska Natives") OR AB (Indigenous OR Aboriginal OR Maori OR “Torres Strait Islander” OR “First Nation” OR Metis OR Inuit OR Inuk OR Innu OR “American Indian” OR “Alaska Native” OR “Native Hawaiian” OR “Pacific Islander” OR Sami OR “Two Spirit”)

S8: AB (Immigrant* OR Refugee OR Undocumented OR Migrant* OR Asylum) OR (MH "Emigrants and Immigrants")

S9: (MH "Jews") OR (MH “Islam”) OR ( AB (Jew* OR Judaism OR Islam* OR Muslim OR Hindu* OR Sikh* OR Roma OR "religious minorit*")

S10: (MH "Prisoners") OR (MH "Enslaved Persons") OR (MH “Juvenile Delinquency”) OR (AB (Inmate* OR Offender OR Incarcerated OR Incarceration OR Prisoner OR detainee))

S11: AB (police OR "law enforcement" OR prison OR correction* OR justice OR court) N4 (brutal* OR harass* OR bias OR shoot* OR beat* OR violen* OR defund* OR decarcerat* OR carding OR frisk* OR militari*)

S12: AB (jail OR prison* OR detention OR incarcer* OR correctional OR corrections OR custody OR arrest OR bail OR frisk* OR carding) OR (MH "Prisons") OR (MH "Capital Punishment") OR (MH “Detention Center”)

S13: (MH "Police") OR (MH "Law Enforcement") OR (MH "Legislation, Drug") OR (MH "Legal Services") OR (MH "Criminal Law") OR (MH "International Law") OR (MH "Supreme Court Decisions") OR (MH "Judicial Role") OR (MH "Human Rights Abuses") OR AB ("criminal justice" OR court OR law OR enforcement)

S14: AB ((raci* OR ethnic*) N3 (structural OR institutional OR bias*))

S15: AB (“Minority stress” OR “Social stressor” OR microaggression OR discriminat* OR segregat* OR Redlining OR “White supremac*” OR Racism OR Racist OR “Institutional Racism” OR Colonial* OR Decoloni* OR Fascis* OR Islamophobi* OR “Anti-Semiti*”)

S16: (MH "Prejudice") OR (MH "Xenophobia") OR (MH "Race Factors") OR (MH "Race Relations") OR (MH "Social Segregation") OR (MH "Racism") OR (MH "Desegregation") OR (MH “Social Discrimination”) OR (MH "Colonialism") OR (MH "Fascism") OR (MH "Apartheid")

S17: AB ("overrepresentation" OR "over-representation" OR "over representation")

S18: (S1 OR S2 OR S3 OR S4 OR S5 OR S6 OR S7 OR S8 OR S9)

S19: S18 AND S11

S20: S18 AND S10 AND S17

S21: S18 AND (S12 OR 13) AND (S14 OR S15 OR S16)

S22: S18 AND AB ((Determinants OR disparit* OR inequit* OR bias* OR marginali*) N3 (Structural OR Institutional))

S23: ( S18 AND (S10 OR S12 OR S13) ) AND ( (MH "Social Determinants of Health") OR (MH "Health Equity") OR (MH "Health Status Disparities") OR (MH "Healthcare Disparities") OR (MH "Health Status") OR (MH "Health Status Indicators") OR (MH "Social Responsibility") OR (MH "Social Conditions") OR (MH “Social Marginalization”) OR (MH "Quality of Life") OR TI (determinants OR disparit* OR equit* OR equalit* OR inequit* OR inequal* OR unequal* OR disadvantage* OR upstream OR barriers OR facilitators) )

S24: S18 AND (S10 OR S12 OR S13) AND ((MH "Civil Rights") OR (MH "Right to Health") OR (MH "Human Rights") OR (MH "Women's Rights") OR (MH "Social Justice") OR (MH "Freedom"))

S25: ( S18 AND (S10 OR S12 OR S13)) AND ((MH "Government") OR (MH "Local Government") OR (MH "State Government") OR (MH "Government Agencies") OR (MH "Federal Government") OR (MH "Government Programs") OR (MH "Government Regulation") OR (MH "Public Policy") OR (MH "Legislation as Topic") OR (MH "Social Control, Formal") OR (MH "Policy Making") OR (MH "Mandatory Programs+") OR (MH "Fiscal Policy") OR (MH “Taxes”) OR (MH "Financing, Government") OR (MH "Antitrust Laws") OR (MH "Privatization") OR (MH "Public Nondiscrimination Policies") OR AB (policy OR policies OR "by-law" OR legislation OR tax* OR law OR legal OR statute OR redlinin* OR budget OR funding OR funds OR gerrymander*) OR AB ((govern* OR politic*) N3 (local OR municipal* OR state OR provinc* OR federal OR program* OR policy OR policies OR legislat* OR law OR will OR decision*)))

S26: S18 AND (S14 OR S15 OR S16) AND ((MH "Government") OR (MH "Local Government") OR (MH "State Government") OR (MH "Government Agencies") OR (MH "Federal Government") OR (MH "Government Programs") OR (MH "Government Regulation") OR (MH "Public Policy") OR (MH "Legislation as Topic") OR (MH "Social Control, Formal") OR (MH "Policy Making") OR (MH "Mandatory Programs+") OR (MH "Fiscal Policy") OR (MH “Taxes”) OR (MH "Financing, Government") OR (MH "Antitrust Laws") OR (MH "Privatization") OR (MH "Public Nondiscrimination Policies") OR AB (policy OR policies OR "by-law" OR legislation OR tax* OR law OR legal OR statute OR redlinin* OR budget OR funding OR funds OR gerrymander*) OR AB ((govern* OR politic*) N3 (local OR municipal* OR state OR provinc* OR federal OR program* OR policy OR policies OR legislat* OR law OR will OR decision*)))

S27: ( S18 AND (S14 OR S15 OR S16) ) AND ( (MH "Civil Rights") OR (MH "Right to Health") OR (MH "Human Rights") OR (MH "Women's Rights") OR (MH "Social Justice") OR (MH "Freedom") )

S28: ( ( S18 AND (S14 OR S15 OR S16) ) AND ( ( (MH "Social Determinants of Health") OR (MH "Health Equity") OR (MH "Health Status Disparities") OR (MH "Healthcare Disparities") OR (MH "Health Status") OR (MH "Health Status Indicators") OR (MH "Social Responsibility") OR (MH "Social Conditions") OR (MH “Social Marginalization”) OR (MH "Quality of Life") OR TI (determinants OR disparit* OR equit* OR equalit* OR inequit* OR inequal* OR unequal* OR disadvantage* OR upstream OR barriers OR facilitators) ) )

S29: S19 OR S20 OR S21 OR S22 OR S23 OR S24 OR S25 OR S26 OR S27 OR S28

S30: S19 OR S20 OR S21 OR S22 OR S23 OR S24 OR S25 OR S26 OR S27 OR S28

S31: AB (Austri* OR Belgium OR Belgian OR Bulgaria* OR Croatia* OR Cyprus OR Czech* OR Denmark OR Danish OR Estonia* OR Finland OR Finnish OR France OR French OR German* OR Greece OR Greek OR Hungar* OR Ireland OR Irish OR Italy OR Italian OR Latvia* OR Lithuania* OR Luxembourg OR Malta OR Netherlands OR Holland OR Poland OR Polish OR Portugal OR Portuguese OR Romania* OR Slovakia* OR Slovenia* OR Spain OR Sweden OR England OR “Northern Ireland” OR Wales OR Welsh OR Scotland OR Scottish OR Argentin* OR Australia* OR Brazil* OR Canad* OR China OR Chinese OR France OR French OR German* OR India OR Indian OR Indonesia* OR Italian OR Italy OR Japan* OR “South Korea” OR Korean OR Mexic* OR Russia* OR “Saudi Arabia*” OR “South Africa*” OR Turkey OR Turkish OR “United Kingdom” OR “United States” OR America* OR “European Union” OR "New Zealand" OR Norway OR Norwegian)

S32: S29 AND S31

S33: S30 AND S31

S34: S33 NOT S32

**Search 3 – Focused on the intersections between climate change, environmental disasters that cause mass casualties, racism, racialized populations and government policies or capitalism.**

S1: (MH "Disasters") OR (MH "Natural Disasters+") OR (MH "Mass Casualty Incidents") OR (MH "Emergencies") OR (MH "Floods") OR (MH "Cyclonic Storms") OR (MH "Droughts") OR (MH "Sea Level Rise") OR (MH "Fires") OR (MH "Wildfires") OR (MH "Weather+") OR AB (disaster OR "public health emergency" OR "mass casualty" OR earthquake OR tornado OR storm OR fire OR hurricane OR typhoon)

S2: MH "Ecosystem+") OR (MH "Climate+") OR (MH "Environment") OR (MH "Natural Resources") OR (MH "Wilderness") OR (MH "Soil+") OR (MH "Farms") OR (MH "Organic Agriculture") OR (MH "Agriculture") OR (MH "Livestock") OR (MH "Crops, Agricultural") OR (MH "Plants") OR (MH "Trees") OR (MH "Plants, Edible") OR (MH "Vegetables") OR (MH "Edible Grain") OR (MH "Crop Production") OR (MH "Gardens") OR (MH "Horticulture") OR (MH "Gardening") OR (MH "Parks, Recreational") OR AB (environment* OR crops OR agricultur* OR "agri-food" OR farm* OR soil OR plants OR vegetation OR parks OR park OR “green space” OR climate OR ecolog* OR ecosystem* OR crop* OR natural* OR land OR water OR wilderness OR “natural resource”)

S3: (MH "Water Resources") OR (MH "Water") OR (MH "Fresh Water+") OR (MH "Seawater") OR (MH "Oceans and Seas+") OR (MH "Fisheries") OR (MH "Bathing Beaches") OR (MH "Water Cycle") OR (MH "Rain+") OR AB (water OR lakes OR ocean OR sea OR pond OR fisheries OR fishery OR beach* OR river)

S4: (MH "Water Wells") OR (MH "Drinking Water") OR (MH "Water Supply") OR AB ((well OR cistern OR drinking OR ground OR municipal*) N5 water)

S5: MH “Environmental Pollution”) OR (MH "Water Pollution+") OR (MH "Air Pollution") OR (MH "Petroleum Pollution") OR (MH "Air Pollution, Radioactive") OR (MH "Biodegradation, Environmental") OR (MH "Water Pollutants+") OR (MH "Environmental Pollutants") OR (MH "Soil Pollutants+") OR (MH "Air Pollutants+") OR (MH "Waste Management+") OR (MH "Sanitation+") OR (MH "Refuse Disposal+") OR (MH "Recycling") OR (MH "Waste Disposal, Fluid") OR (MH "Waste Water") OR (MH "Waste Products+") OR AB (pollut* OR waste OR disposal OR refuse OR recycl* OR footprint OR endanger* OR destruct* OR contaminat* OR poison* OR "fossil fuel" OR pipeline)

S6: (MH "Climate Change") OR (MH "Carbon Footprint") OR (MH "Greenhouse Effect") OR (MH "Global Warming") OR (MH "Endangered Species")

S7: (MH "Prejudice") OR (MH "Race Factors") OR (MH "Race Relations") OR (MH "Social Segregation") OR (MH "Racism") OR (MH "Desegregation") OR (MH “Social Discrimination”) OR (MH "Colonialism") OR (MH "Fascism") OR (MH "Apartheid") OR AB (discriminat* OR segregat* OR Redlining OR “White supremac*” OR Racism OR Racist OR “Institutional Racism” OR Colonial* OR Decoloni* OR Fascis*)

S8: AB ((raci* OR ethnic*) N3 (structural OR institutional OR bias*))

S9: (MH "Social Determinants of Health") OR (MH "Health Equity") OR (MH "Health Status Disparities") OR (MH "Health Status") OR (MH "Social Responsibility") OR (MH "Social Conditions") OR (MH “Social Marginalization”) OR (MH "Quality of Life") OR (MH "Civil Rights") OR (MH "Right to Health") OR (MH "Human Rights") OR (MH "Women's Rights") OR (MH "Social Justice")

S10: ( AB ((Determinants OR disparit* OR inequit* OR bias* OR marginali*) N3 (Structural OR Institutional)) ) OR ( TI (determinants OR disparit* OR equit* OR equalit* OR inequit* OR inequal* OR unequal* OR disadvantage* OR upstream OR barriers OR facilitators) )

S11: (MH "Government") OR (MH "Local Government") OR (MH "State Government") OR (MH "Government Agencies") OR (MH "Federal Government") OR (MH "Government Programs") OR (MH "Government Regulation") OR (MH "Public Policy") OR (MH "Environmental Policy") OR (MH "Health Policy") OR (MH "Mandatory Testing") OR (MH "Mandatory Programs") OR (MH "Mandatory Reporting") OR (MH "Law Enforcement") OR (MH "Liability, Legal") OR (MH "International Law") OR (MH "Policy Making") OR (MH "Legislation as Topic") OR (MH "Threshold Limit Values") OR (MH "Legislation, Food") OR (MH "Fiscal Policy") OR (MH “Taxes”) OR (MH "Financing, Government") OR (MH "Privatization") OR (MH "Environment and Public Health") OR (MH "Conservation of Natural Resources") OR (MH "Sustainable Development") OR (MH "Environmental Restoration and Remediation") OR (MH "Conservation of Water Resources") OR (MH "Conservation of Energy Resources") OR (MH "Disaster Planning+")

S12: AB (Justice OR rights OR Protecti* OR legislat* OR law OR "by-law" OR limit* OR threshold OR conserv* OR policy OR policies OR tax* OR statute OR budget OR funding OR funds OR gerrymander* OR mitigat*)

S13: AB ((govern* OR politic*) N3 (local OR municipal* OR state OR provinc* OR federal OR program* OR policy OR policies OR legislat* OR law OR will OR decision*))

S14: (MH "Indigenous Peoples") OR (MH "American Native Continental Ancestry Group") OR (MH "Oceanic Ancestry Group") OR (MH "Indians, North American") OR (MH "Indians, South American") OR (MH "Indians, Central American") OR (MH "Inuits") OR (MH "Alaska Natives") OR AB (Indigenous OR Aboriginal OR Maori OR “Torres Strait Islander” OR “First Nation” OR Metis OR Inuit OR Inuk OR Innu OR “American Indian” OR “Alaska Native” OR “Native Hawaiian” OR “Pacific Islander” OR Sami OR “Two Spirit”)

S15: (MH "African Americans") OR (MH "African Continental Ancestry Group") OR AB (Black OR "African-American" OR African OR Caribbean OR Afro* OR "BAME" OR “Black, Asian and minority ethnicity”) OR AB ("Mixed-race" OR biracial OR "bi-racial" OR "multiracial" OR "multi-racial"OR raciali*) OR (MH "Arabs") OR (AB (“Middle East*” OR “North African” OR “Arabic-speaking” OR “English as a second language”) OR (MH "Hispanic Americans") OR (MH "Mexican Americans") OR AB (Latina OR Latino OR Latinx OR Hispanic OR “Spanish-speaking” “English as a second language”) OR (MH "Asian Americans") OR (MH "Asian Continental Ancestry Group") OR AB (“East Asian” OR “South Asian” OR “Chinese-speaking” OR “Cantonese-speaking” OR “Mandarin-speaking” OR “English as a second language”) OR (MH "Ethnic Groups") OR AB ( (Ethnic* OR ancestry) )

16: (MH "Vulnerable Populations")

S17: AB (Immigrant* OR Refugee OR Undocumented OR Migrant* OR Asylum OR “Asylum Seek*”) OR (MH "Emigrants and Immigrants")

S18: AB (energy OR water OR food) N3 (secur* OR poverty)

S19: AB (contaminat* OR poison* OR pollut*) N3 (mercury OR arsenic OR "heavy metal") AND (S3 OR S4)

S20: AB (“green new deal” OR “Paris Agreement”)

S21: (S18 OR S19) AND (S7 OR S8) AND (S14 OR S15 OR S16 OR S17)

S22: (S18 OR S19) AND (S14 OR S15 OR S16 OR S17) AND (S9 OR S10)

S23: (S18 OR S19) AND (S9 OR S10) AND (S11 OR S12 OR S13)

S24: (S1 OR S2 OR S3 OR S4) AND (S5 OR S6) AND (S9 OR S10) AND (S14 OR S15 OR S16 OR S17)

S25: (S1 OR S2 OR S3 OR S4) AND (S5 OR S6) AND (S9 OR S10)

S26: (S1 OR S2 OR S3 OR S4) AND (S5 OR S6) AND (S9 OR S10) AND (S11 OR S12 OR S13)

S27: (S1 OR S2 OR S3 OR S4) AND (S5 OR S6) AND (S7 OR S8)

S28: (S1 OR S2 OR S3 OR S4) AND (S5 OR S6) AND (S7 OR S8) AND (S14 OR S15 OR S16 OR S17)

S29: (S1 OR S2 OR S3 OR S4) AND (S5 OR S6) AND (S11 OR S12 OR S13) AND (S14 OR S15 OR S16 OR S17)

S30: S20 OR S22 OR S23 OR S24 OR S25 OR S26 OR S27 OR S28 OR S29

S31: AB (Argentin* OR Australia* OR Austri* OR Belgium OR Belgian OR Brazil OR Bulgaria* OR Canad* OR China OR Chinese OR Croatia* OR Cyprus OR Czech* OR Denmark OR Danish OR Estonia* OR Finland OR Finnish OR France OR French OR German* OR Greece OR Greek OR Hungar* OR Ireland OR Irish OR Italy OR Italian OR India OR Indian OR Indonesia* OR Latvia* OR Lithuania* OR Luxembourg OR Malta OR Mexic* OR Netherlands OR Holland OR Poland OR Polish OR Portugal OR Portuguese OR Romania* OR Slovakia* OR Slovenia* OR Spain OR Sweden OR England OR “Northern Ireland” OR Wales OR Welsh OR Scotland OR Scottish OR Japan* OR “South Korea” OR Korean OR Russia* OR “Saudi Arabia*” OR “South Africa*” OR Turkey OR Turkish OR “United Kingdom” OR “United States” OR America* OR “European Union” OR "New Zealand" OR Norway OR Norwegian) OR (MH "Austria") OR (MH "Argentina") OR (MH "Australia+") OR (MH "Belgium") OR (MH "Brazil") OR (MH "Bulgaria") OR (MH "Canada+") OR (MH "China+") OR (MH "Czech Republic") OR (MH "Croatia") OR (MH "Cyprus") OR (MH "Denmark+") OR (MH "European Union") OR (MH "Europe") OR (MH "England+") OR (MH "Estonia") OR (MH "Finland") OR (MH "France+") OR (MH "Greece") OR (MH "Germany") OR (MH "Hong Kong") OR (MH "Hungary") OR (MH "Italy+") OR (MH "Ireland") OR (MH "India") OR (MH "Indonesia") OR (MH "Japan+") OR (MH "Korea") OR (MH "Republic of Korea") OR (MH "Latvia") OR (MH "Lithuania") OR (MH "Malta") OR (MH "Netherlands") OR (MH "Norway+") OR (MH "New Zealand") OR (MH "Northern Ireland") OR (MH "North America") OR (MH "Portugal") OR (MH "Poland") OR (MH "Romania") OR (MH "Slovenia") OR (MH "Slovakia") OR (MH "Saudi Arabia") OR (MH "Scotland") OR (MH "Switzerland") OR (MH "Spain") OR (MH "Scandinavian and Nordic Countries") OR (MH "Sweden") OR (MH "Wales") OR (MH "Turkey") OR (MH "South Africa") OR (MH "Russia") OR (MH "United Kingdom") OR (MH "United States+") OR (MH "Wales")

S32: S30 AND S31

S33: S30 AND S31

S34: S32 NOT S33
