# Supplementary material for: Structural interventions that affect racial inequities and their impact on population health outcomes: a systematic review
Source: BMC Public Health. 2022 Nov 24;22:2162. doi: 10.1186/s12889-022-14603-w (PMC9685079; doi:10.1186/s12889-022-14603-w)
Supplement: Supplementary file 2 — Additional file 2. Excluded Studies. [file 12889_2022_14603_MOESM2_ESM.docx]

Additional file 2: Excluded Studies

1. Ahmed S, Fielding D. Changes in maternity leave coverage: Implications for fertility, labour force participation and child mortality. 2019;241:112573.

2. Akbulut-Yuksel M. Do legal school leaving rules still affect schooling and earnings? Social science research. 2017;61:195-205.

3. Akhter N, Bambra C, Mattheys K, Warren J, Kasim A. Inequalities in mental health and well-being in a time of austerity: Follow-up findings from the Stockton-on-Tees cohort study. SSM - Population Health. 2018;6:75-84.

4. Andersen M, Dusheiko M, Grassi S. The effect of health insurance subsidies on mortality for low income individuals. 2015.

5. Andrade FCD, Kramer KZ, Greenlee A, Williams AN, Mendenhall R. Impact of the Chicago Earned Income Tax Periodic Payment intervention on food security. 2019;16:100993-.

6. Andrade MV, Noronha KVMdS, Queiroz Barbosa AC, Souza MN, Calazans JA, Carvalho LRd, et al. Family health strategy and equity in prenatal care: a population based cross-sectional study in Minas Gerais, Brazil. Int J Equity Health. 2017;16(1):24.

7. Andrea SB, Messer LC, Marino M, Goodman JM, Boone-Heinonen J. A nationwide investigation of the impact of the tipped worker subminimum wage on infant size for gestational age. 2020;133:106016.

8. Andreyeva E, Ukert B. The impact of the minimum wage on health. 2018;18(4):337-75.

9. Antonipillai V, Baumann A, Hunter A, Wahoush O, O'Shea T. Health Inequity and "Restoring Fairness" Through the Canadian Refugee Health Policy Reforms: A Literature Review. Journal of immigrant and minority health. 2018;20(1):203-13.

10. Aran MA, Aktakke N, Gurol-Urganci I, Atun RA. Maternal and Child Health in Turkey Through the Health Transformation Program (2003-2008). 2015(1501).

11. Arno PS, House JS, Viola D, Schechter C. Social security and mortality: the role of income support policies and population health in the United States. 2011;32(2):234-50.

12. Arno PS, Wicks-Lim J. The earned income tax credit’s impact on health. 2015:25-.

13. Asaria M, Ali S, Doran T, Ferguson B, Fleetcroft R, Goddard M, et al. How a universal health system reduces inequalities: lessons from England. 2016;70(7):637-43.

14. Atun R, Aydın S, Chakraborty S, Sümer S, Aran M, Gürol I, et al. Universal health coverage in Turkey: enhancement of equity. The Lancet. 2013;382(9886):65-99.

15. Averett S, Wang Y. The effect of the EITC payment expansion on maternal smoking. 2012(6680).

16. Averett S, Wang Y. Effects of Higher EITC Payments on Children’s Health, Quality of Home Environment, and Noncognitive Skills. Public Finance Review. 2018;46(4):519-57.

17. Ayyagari P. Evaluating the impact of social security benefits on health outcomes among the elderly. 2015.

18. Baird S, Ferreira FHG, Özler B, Woolcock M. Relative Effectiveness of Conditional and Unconditional Cash Transfers for Schooling Outcomes in Developing Countries: A Systematic Review. Campbell Systematic Reviews. 2013;9(1):1-124.

19. Baltagi BH, Yen Y. Welfare reform and children's health. 2016;25(3):277-91.

20. Barr B, Bambra C, Whitehead M. The impact of NHS resource allocation policy on health inequalities in England 2001-11: longitudinal ecological study. BMJ. 2014;348(may27 6):g3231-g.

21. Barr B, Higgerson J, Whitehead M. Investigating the impact of the English health inequalities strategy: time trend analysis. 2017;358.

22. Barr BR. The mental health impact of recession and welfare reform in England between 2008 and 2013. 2015.

23. Bastagli F, Hagen-Zanker J, Harman L, Barca V, Sturge G, Schmidt T. The impact of cash transfers: a review of the evidence from low-and middle-income countries. 2019;48(3):569.

24. Batra A, Hamad R. Short-term effects of the earned income tax credit on children's physical and mental health. 2021.

25. Baughman RA, Duchovny N. State earned income tax credits and the production of child health: Insurance coverage, utilization, and health status. 2016;69(1):103-.

26. Beckfield J, Morris KA, Bambra C. How social policy contributes to the distribution of population health: the case of gender health equity. Scandinavian journal of public health. 2018;46(1):6-17.

27. Beenackers MA, van Lenthe FJ, Groeniger JO, Nusselder WJ, from Erasmus JPM. Effective interventions to reduce socioeconomic inequality in health2016.

28. Belenky N, Pence BW, Cole SR, Dusetzina SB, Edmonds A, Oberlander J, et al. Impact of Medicare Part D on mental health treatment and outcomes for dual eligible beneficiaries with HIV. AIDS Care. 2019;31(4):505-12.

29. Bhatt CB, Beck-Sague CM. Medicaid Expansion and Infant Mortality in the United States. 2018;108(4):565-7.

30. Black AP, Brimblecombe J, Eyles H, Morris P, Vally H. Food subsidy programs and the health and nutritional status of disadvantaged families in high income countries: a systematic review. 2012;12(1):1099.

31. Boertien D, Vignoli D. Legalizing Same-Sex Marriage Matters for the Subjective Well-being of Individuals in Same-Sex Unions. 2019;56(6):2109-21.

32. Boudreaux MH, Dagher RK, Lorch SA. The Association of Health Reform and Infant Health: Evidence from Massachusetts. 2018;53(4):2406-25.

33. Boyd-Swan C, Herbst CM, Ifcher J, Zarghamee H. The Earned Income Tax Credit, Health, and Happines. 2012.

34. Boyd-Swan C, Herbst CM, Ifcher J, Zarghamee H. The earned income tax credit, mental health, and happiness. 2016;126:18-38.

35. Brady D, Burroway R. Targeting, universalism, and single-mother poverty: a multilevel analysis across 18 affluent democracies. Demography. 2012;49(2):719-46.

36. Breysse J, Jacobs DE, Weber W, Dixon S, Kawecki C, Aceti S, et al. Health outcomes and green renovation of affordable housing. Public health reports (Washington, DC : 1974). 2011;126:64-75.

37. Brown CC, Moore JE, Felix HC, Stewart MK, Bird TM, Lowery CL, et al. Association of State Medicaid Expansion Status With Low Birth Weight and Preterm Birth. JAMA. 2019;321(16):1598.

38. Brown EM, Tarasuk V. Money speaks: Reductions in severe food insecurity follow the Canada Child Benefit. Preventive medicine. 2019;129:105876.

39. Brown HS, Wilson KJ, Angel JL. Mexican Immigrant Health: Health Insurance Coverage Implications. Journal of health care for the poor and underserved. 2015;26(3):990-1004.

40. Browne J, Lock M, Walker T, Egan M, Backholer K. Effects of food policy actions on Indigenous Peoples’ nutrition-related outcomes: a systematic review. 2020;5(8):e002442-e.

41. Brownell MD, Chartier MJ, Nickel NC, Chateau D, Martens PJ, Sarkar J, et al. Unconditional Prenatal Income Supplement and Birth Outcomes. Pediatrics. 2016;137(6).

42. Bruening M, McClain D, Moramarco M, Reifsnider E. The role of SNAP in home food availability and dietary intake among WIC participants facing unstable housing. 2017;34(3):219-28.

43. Bullinger LR. The Effect of Minimum Wages on Adolescent Fertility: A Nationwide Analysis. American journal of public health. 2017;107(3):447-52.

44. Campbell MA, Hunt J, Scrimgeour DJ, Davey M, Jones V. Contribution of Aboriginal Community-Controlled Health Services to improving Aboriginal health: an evidence review. 2018;42(2):218-26.

45. Canning PM, Frizzell LM, Courage ML. Birth outcomes associated with prenatal participation in a government support programme for mothers with low incomes. Child: Care, Health and Development. 2010;36(2):225-31.

46. Carlson S, Keith-Jennings B. SNAP is linked with improved nutritional outcomes and lower health care costs. 2018:1.

47. Carpenter CS, Eppink ST, Gonzales G, McKay T. Effects of Access to Legal Same‐Sex Marriage on Marriage and Health %J Journal of Policy Analysis and Management.

48. Cesur R, Güneş PM, Tekin E, Ulker A. The value of socialized medicine: The impact of universal primary healthcare provision on mortality rates in Turkey. 2017;150:75-93.

49. Chilenski SM, Frank J, Summers N, Lew D. Public Health Benefits 16 Years After a Statewide Policy Change: Communities That Care in Pennsylvania. Prev Sci. 2019;20(6):947-58.

50. Chriqui JF, Leider J, Thrun E, Nicholson LM, Slater SJ. Pedestrian-oriented zoning is associated with reduced income and poverty disparities in adult active travel to work, United States. Preventive medicine. 2017;95:S126-S33.

51. Cluver LD, Orkin FM, Boyes ME, Sherr L. Cash plus care: social protection cumulatively mitigates HIV-risk behaviour among adolescents in South Africa. AIDS. 2014;28(Supplement 3):S389-S97.

52. Cluver LD, Orkin MF, Yakubovich AR, Sherr L. Combination social protection for reducing HIV-risk behavior amongst adolescents in South Africa. 2016;72(1):96-.

53. Collin DF, Shields-Zeeman LS, Batra A, Vable AM, Rehkopf DH, Machen L, et al. Short-term effects of the earned income tax credit on mental health and health behaviors. 2020;139:106223-.

54. Cook A, Stype A. Medicaid expansion and infant mortality: the (questionable) impact of the Affordable Care Act. 2021;75(1):10-5.

55. Cook K, Davis E, Smyth P, McKenzie H. The quality of life of single mothers making the transition from welfare to work. Women & health. 2009;49(6):475-90.

56. Correia LL, Rocha HAL, Leite ÁJM, Cavalcante e Silva A, Campos JS, Machado MMT, et al. The relation of cash transfer programs and food insecurity among families with preschool children living in semiarid climates in Brazil. 2018;26(1):53-62.

57. Courtemanche C, Marton J, Ukert B, Yelowitz A, Zapata D. Effects of the Affordable Care Act on Health Care Access and Self-Assessed Health After 3 Years. 2018;55:46958018796361.

58. Courtin E, Aloisi K, Miller C, Allen HL, Katz LF, Muennig P. The Health Effects Of Expanding The Earned Income Tax Credit: Results From New York City: Study examines the health effects of the New York City Paycheck Plus program that increases the Earned Income Tax Credit for low-income Americans without dependent c. 2020;39(7):1149-56.

59. Creedon TB, Cook BL. Access To Mental Health Care Increased But Not For Substance Use, While Disparities Remain. Health Affairs. 2016;35(6):1017-21.

60. Crookes DM, Stanhope KK, Kim YJ, Lummus E, Suglia SF. Federal, State, and Local Immigrant-Related Policies and Child Health Outcomes: a Systematic Review. 2021:1-11.

61. Cubanski J, Neuman P. Medicare doesn't work as well for younger, disabled beneficiaries as it does for older enrollees. Health affairs (Project Hope). 2010;29(9):1725-33.

62. Currie J, Castillo MG, Adekanmbi V, Barr B, Flaherty M. Evaluating effects of recent changes in NHS resource allocation policy on inequalities in amenable mortality in England, 2007–2014: time-series analysis. 2019;73(2):162-7.

63. Cylus J, Glymour MM, Avendano M. Health effects of unemployment benefit program generosity. 2015;105(2):317-23.

64. Daw JR, Sommers BD. Association of the Affordable Care Act dependent coverage provision with prenatal care use and birth outcomes. 2018;319(6):579.

65. Deb P, Gregory CA. Heterogeneous impacts of the supplemental nutrition assistance program on food insecurity. 2018;173:55.

66. Delaruelle K, van de Werfhorst H, Bracke P. Do comprehensive school reforms impact the health of early school leavers? results of a comparative difference-in-difference design. 2019;239:112542.

67. Dunlop AL, Joski P, Strahan AE, Sierra E, Adams EK. Postpartum Medicaid Coverage and Contraceptive Use Before and After Ohio's Medicaid Expansion Under the Affordable Care Act. 2020;30(6):426.

68. Edmonds AT, Moe CA, Adhia A, Mooney SJ, Rivara FP, Hill HD, et al. The Earned Income Tax Credit and Intimate Partner Violence. 2021:0886260521997440-.

69. Eguia E, Cobb AN, Kothari AN, Molefe A, Afshar M, Aranha GV, et al. Impact of the Affordable Care Act (ACA) Medicaid Expansion on Cancer Admissions and Surgeries. Annals of Surgery. 2018;268(4):584-90.

70. Eliason EL. Adoption of Medicaid expansion is associated with lower maternal mortality. 2020;30(3):147.

71. Esteves RJF. The quest for equity in Latin America: a comparative analysis of the health care reforms in Brazil and Colombia. International journal for equity in health. 2012;11:6.

72. Fan M, Jin Y. The Supplemental Nutrition Assistance Program and Childhood Obesity in the United States: Evidence from the National Longitudinal Survey of Youth 1997. 2015;1(4):432.

73. Farrants K. Recommodification and the social determinants of health: unemployment benefits, pensions and health inequalities in Sweden and England, 1991-2011. Journal of public health (Oxford, England). 2017;39(4):661-7.

74. Ferrazzi P, Krupa T. "Symptoms of something all around us": Mental health, Inuit culture, and criminal justice in Arctic communities in Nunavut, Canada. Social science & medicine (1982). 2016;165:159-67.

75. Ford KJ, Lourenço BH, Cobayashi F, Cardoso MA. Health outcomes of the Bolsa Família program among Brazilian Amazonian children. Revista de saude publica. 2020;54:2.

76. Foster EM, Jiang M, Gibson-Davis CM. The effect of the WIC program on the health of newborns. Health services research. 2010;45(4):1083-104.

77. Gai Y, Marthinsen J. Medicaid Expansion, HIV Testing, and HIV-Related Risk Behaviors in the United States, 2010-2017. American journal of public health. 2019;109(10):1404-12.

78. García-Gómez P, Gielen AC. Mortality effects of containing moral hazard: Evidence from disability insurance reform. Health economics. 2018;27(3):606-21.

79. Goodyear-Smith F, Ashton T. New Zealand health system: universalism struggles with persisting inequities. Lancet (London, England). 2019;394(10196):432-42.

80. Gotanda H, Kominski GF, Elashoff D, Tsugawa Y. Association Between the ACA Medicaid Expansions and Changes in Cardiovascular Risk Factors Among Low-Income Individuals. 2021:1.

81. Granados JAT. Politics and health in eight European countries: a comparative study of mortality decline under social democracies and right-wing governments. Social science & medicine (1982). 2010;71(5):841-50.

82. Graves JA, Hatfield LA, Blot W, Keating NL, McWilliams JM. Medicaid Expansion Slowed Rates Of Health Decline For Low-Income Adults In Southern States: An analysis of the impact of Medicaid expansion on the self-reported health of low-income older nonelderly adults living in the South. 2020;39(1):67.

83. Griffith R, von Hinke S, Smith S. Getting a healthy start: The effectiveness of targeted benefits for improving dietary choices. 2018;58:176-87.

84. Grinspun A. No small change: The multiple impacts of the Child Support Grant on child and adolescent well-being. 2016:44-.

85. Hadley A, Ingham R, Chandra-Mouli V. Implementing the United Kingdom's ten-year teenage pregnancy strategy for England (1999-2010): How was this done and what did it achieve? Reproductive health. 2016;13(1):139.

86. Haegerich TM, Jones CM, Cote P-O, Robinson A, Ross L. Evidence for state, community and systems-level prevention strategies to address the opioid crisis. Drug and alcohol dependence. 2019;204:107563.

87. Hafner L, Lochner B. Do minimum wages improve self-rated health? Evidence from a natural experiment. 2019.

88. Hamad R, Batra A, Karasek D, LeWinn KZ, Bush NR, Davis RL, et al. The Impact of the Revised WIC Food Package on Maternal Nutrition During Pregnancy and Postpartum. 2019;188(8):1493-502.

89. Hamad R, Rehkopf DH. Poverty and Child Development: A Longitudinal Study of the Impact of the Earned Income Tax Credit. 2016;183(9):775-84.

90. Harvey SM, Gibbs S, Oakley L, Luck J, Yoon J. Medicaid expansion and neonatal outcomes in Oregon. 2020.

91. Hawkins SS, Baum CF. Impact of state cigarette taxes on disparities in maternal smoking during pregnancy. 2014;104(8):1464.

92. Heflin CM, Ingram SJ, Ziliak JP. The Effect Of The Supplemental Nutrition Assistance Program On Mortality. 2019;38(11):1807.

93. Herbst CM, Tekin E. CHILD CARE SUBSIDIES, MATERNAL HEALTH, AND CHILD-PARENT INTERACTIONS: EVIDENCE FROM THREE NATIONALLY REPRESENTATIVE DATASETS: CHILD CARE SUBSIDIES AND MATERNAL HEALTH. Health Econ. 2014;23(8):894-916.

94. Herd P, Schoeni RF, House JS. Upstream Solutions: Does the Supplemental Security Income Program Reduce Disability in the Elderly? Milbank Quarterly. 2008;86(1):5-45.

95. Hernandez EM, Vuolo M, Frizzell LC, Kelly BC. Moving upstream: The effect of tobacco clean air restrictions on educational inequalities in smoking among young adults. 2019;56(5):1693.

96. Hillier-Brown F, Thomson K, McGowan V, Cairns J, Eikemo TA, Gil-Gonzále D, et al. The effects of social protection policies on health inequalities: Evidence from systematic reviews. Scand J Public Health. 2019;47(6):655-65.

97. Hoff BMa. The association between state Medicaid expansion and human papillomavirus vaccination. 2020;38(38):5963.

98. Hone T, Rasella D, Barreto ML, Majeed A, Millett C. Association between expansion of primary healthcare and racial inequalities in mortality amenable to primary care in Brazil: A national longitudinal analysis. PLoS Med. 2017;14(5):e1002306.

99. Hudak KM, Racine EF. The supplemental nutrition assistance program and child weight status: A review. 2019;56(6):882-93.

100. Hyseni L, Atkinson M, Bromley H, Orton L, Lloyd-Williams F, McGill R, et al. The effects of policy actions to improve population dietary patterns and prevent diet-related non-communicable diseases: scoping review. 2017;71(6):694-711.

101. Imai H, Fujii Y, Fukuda Y, Nakao H, Yahata Y. Health-related quality of life and beneficiaries of long-term care insurance in Japan. Health policy (Amsterdam, Netherlands). 2008;85(3):349-55.

102. Ionescu-Ittu R, Glymour MM, Kaufman JS. A difference-in-differences approach to estimate the effect of income-supplementation on food insecurity. Preventive Medicine. 2015;70:108-16.

103. Johnson AD, Han W-J, Ruhm CJ, Waldfogel J. Child care subsidies and the school readiness of children of immigrants. Child development. 2014;85(6):2140-50.

104. Johnson P, Montgomery M, Ewell P. Federal Food Assistance Programs and Cardiovascular Risk Factors in Low-Income Preschool Children. Journal of community health. 2016;41(3):626-34.

105. Johnston EM, McMorrow S. The Relationship Between Insurance Coverage and Use of Prescription Contraception by Race and Ethnicity: Lessons From the Affordable Care Act. Women's health issues : official publication of the Jacobs Institute of Women's Health. 2020;30(2):73-82.

106. Juarez S, Honkaniemi H, Dunlavy A, Aldridge R, Barreto M, Katikireddi S, et al. Effects of non-healhttargeted policies on migrant health: a systematic review and meta-analysis. Lancet GlobHealth 2019; 7: e420-35.

107. Juárez SP, Honkaniemi H, Dunlavy AC, Aldridge RW, Barreto ML, Katikireddi SV, et al. Effects of non-health-targeted policies on migrant health: a systematic review and meta-analysis. The Lancet Global health. 2019;7(4):e420-e35.

108. Kaestner R, Lee WC. The effect of welfare reform on prenatal care and birth weight. Health economics. 2005;14(5):497-511.

109. Kenney G, Sommers AS, Dubay L. Moving to mandatory Medicaid managed care in Ohio: impacts on pregnant women and infants. Medical care. 2005;43(7):683-90.

110. Khatana SAM, Bhatla A, Nathan AS, Giri J, Shen C, Kazi DS, et al. Association of Medicaid Expansion With Cardiovascular Mortality. 2019;4(7):671-9.

111. Kim S, Kwon S. Has the National Health Insurance improved the inequality in the use of tertiary-care hospitals in Korea? Health policy (Amsterdam, Netherlands). 2014;118(3):377-85.

112. Kimbro RT, Rigby E. Federal food policy and childhood obesity: a solution or part of the problem? 2010;29(3):411-8.

113. Kingsley M, Setodji CM, Pane JD, Shadel WG, Song G, Robertson J, et al. Short-term impact of a flavored tobacco restriction: changes in youth tobacco use in a Massachusetts community. 2019;57(6):741.

114. Ko M, Sanders C, de Guia S, Shimkhada R, Ponce NA. Managing Diversity To Eliminate Disparities: A Framework For Health. Health affairs (Project Hope). 2018;37(9):1383-93.

115. Komro KA, Livingston MD, Markowitz S, Wagenaar AC. The effect of an increased minimum wage on infant mortality and birth weight. 2016;106(8):1514-6.

116. Koyama Y, Fujiwara T, Isumi A, Doi S. Is Japan’s child allowance effective for the well-being of children? A statistical evaluation using data from K-CHILD study. 2020;20(1):1-12.

117. Kronenberg C, Jacobs R, Zucchelli E. The impact of the UK National Minimum Wage on mental health. 2017;3:749-55.

118. Labrecque JA, Kaufman JS. Health profile differences between recipients and non-recipients of the Brazilian Income Transfer Program in a low-income population. Cadernos de saude publica. 2019;35(6):e00141218.

119. Lagarde M, Haines A, Palmer N. The impact of conditional cash transfers on health outcomes and use of health services in low and middle income countries. 2009(4).

120. Larrimore J. Does a higher income have positive health effects? Using the earned income tax credit to explore the income‐health gradient. 2011;89(4):694.

121. Lebihan L, Mao Takongmo CO. Unconditional cash transfers and parental obesity. 2019;224:116-26.

122. Lee H, Porell FW. The Effect of the Affordable Care Act Medicaid Expansion on Disparities in Access to Care and Health Status. 2020;77(5):461-73.

123. Lee LK, Chien A, Stewart A, Truschel L, Hoffmann J, Portillo E, et al. Women's Coverage, Utilization, Affordability, And Health After The ACA: A Review Of The Literature: A literature review of evidence relating to the Affordable Care Act's impact on women's health care and health. 2020;39(3):387.

124. Lenhart O. The impact of minimum wages on population health: evidence from 24 OECD countries. 2017;18(8):1031-9.

125. Lenhart O. Safety Net Against Hunger? The Effects of the Earned Income Tax Credit on Food Insecurity. 2019.

126. Leung CW, Blumenthal SJ, Hoffnagle EE, Jensen HH, Foerster SB, Nestle M, et al. Associations of food stamp participation with dietary quality and obesity in children. 2013;131(3):463-72.

127. Leung CW, Tester JM, Rimm EB, Willett WC. SNAP Participation and Diet-Sensitive Cardiometabolic Risk Factors in Adolescents. 2017;52(2):S127-S37.

128. Levy DT, Mumford EA, Compton C. Tobacco control policies and smoking in a population of low education women, 1992-2002. 2006;60:20-6.

129. Li N, Dachner N, Tarasuk V. The impact of changes in social policies on household food insecurity in British Columbia, 2005-2012. Preventive medicine. 2016;93:151-8.

130. Lichtman-Sadot S, Bell NP. Child Health in Elementary School Following California’s Paid Family Leave Program. Journal of policy analysis and management : [the journal of the Association for Public Policy Analysis and Management]. 2017;36(4):790-827.

131. Liu X, Wong H, Liu K. Outcome-based health equity across different social health insurance schemes for the elderly in China. BMC health services research. 2016;16:9.

132. Loehrer AP, Song Z, Auchincloss HG, Hutter MM. Massachusetts health care reform and reduced racial disparities in minimally invasive surgery. JAMA surgery. 2013;148(12):1116-22.

133. Lundberg O, Yngwe MA, Stjärne MK, Elstad JI, Ferrarini T, Kangas O, et al. The role of welfare state principles and generosity in social policy programmes for public health: an international comparative study. Lancet (London, England). 2008;372(9650):1633-40.

134. Machat S, Shannon K, Braschel M, Moreheart S, Goldenberg SM. Sex workers’ experiences and occupational conditions post-implementation of end-demand criminalization in Metro Vancouver, Canada. Can J Public Health. 2019;110(5):575-83.

135. Malbon E, Carey G, Meltzer A. Personalisation schemes in social care: are they growing social and health inequalities? BMC public health. 2019;19(1):805.

136. Manley J, Slavchevska V. Are cash transfers the answer for child nutrition in sub‐Saharan Africa? A literature review. 2019;37(2):204-24.

137. Marbach M, Hainmueller J, Hangartner D. The long-term impact of employment bans on the economic integration of refugees. Science advances. 2018;4(9):eaap9519.

138. Marchetti-Bowick E. Does Welfare Reduce Mortality? Evidence from the Supplemental Security Income Program. 2017.

139. Margerison CE, Kaestner R, Chen J, MacCallum-Bridges C. Impacts of Medicaid Expansion Prior to Conception on Pre-pregnancy Health, Pregnancy Health, and Outcomes. 2020.

140. Margerison CE, MacCallum CL, Chen J, Zamani-Hank Y, Kaestner R. Impacts of Medicaid expansion on health among women of reproductive age. 2020;58(1):1.

141. Marino M, Angier H, Fankhauser K, Valenzuela S, Hoopes M, Heintzman J, et al. Disparities in Biomarkers for Patients With Diabetes After the Affordable Care Act. Medical care. 2020;58:S31-S9.

142. Markowitz S, Komro KA, Livingston MD, Lenhart O, Wagenaar AC. Effects of state-level earned income tax credit laws in the US on maternal health behaviors and infant health outcomes. 2017;194:67.

143. Markus AR, Krohe S, Garro N, Gerstein M, Pellegrini C. Examining the association between Medicaid coverage and preterm births using 2010–2013 National Vital Statistics Birth Data. 2017;23(1):79.

144. Martinez O, Wu E, Sandfort T, Dodge B, Carballo-Dieguez A, Pinto R, et al. Evaluating the impact of immigration policies on health status among undocumented immigrants: a systematic review. 2015;17(3):947-70.

145. Mathews C, Goga A, Loveday M, Zembe W, Daviaud E, Siegfried N, et al. Moving towards universal health coverage: Strengthening the evidence ecosystem for the South African health system. South African medical journal = Suid-Afrikaanse tydskrif vir geneeskunde. 2019;109(11):8-14.

146. McConville S. Medi-Cal Expansion and Children's Well-Being. 2019.

147. McIntyre L, Kwok C, Emery JCH, Dutton DJ. Impact of a guaranteed annual income program on Canadian seniors’ physical, mental and functional health. 2016;107(2):e176-e82.

148. McMorrow S, Kenney GM, Long SK, Goin DE. Medicaid Expansions from 1997 to 2009 Increased Coverage and Improved Access and Mental Health Outcomes for Low-Income Parents. 2016;51(4):1347-67.

149. Meghea CI, You Z, Raffo J, Leach RE, Roman LA. Statewide Medicaid Enhanced Prenatal Care Programs and Infant Mortality. Pediatrics. 2015;136(2):334-42.

150. Miller S, Johnson N, Wherry LR. Medicaid and mortality: new evidence from linked survey and administrative data. 2019.

151. Miraldo M, Propper C, Williams RI. The impact of publicly subsidised health insurance on access, behavioural risk factors and disease management. Social science & medicine (1982). 2018;217:135-51.

152. Monteiro Andrade LO, de Holanda Cunha Bareta IC, Ferreira Gomes C, Chagas Canuto OM. Public health policies as guides for local public policies: the experience of Sobral-Ceará, Brazil. Promotion & Education. 2005;12(3_suppl):28-31.

153. Muennig P, Vail D, Hakes JK. Can antipoverty programmes save lives? Quasi-experimental evidence from the Earned Income Tax Credit in the USA. 2020;10(8):e037051-e.

154. Narain K, Bitler M, Ponce N, Kominski G, Ettner S. The impact of welfare reform on the health insurance coverage, utilization and health of low education single mothers. Social science & medicine (1982). 2017;180:28-35.

155. Nelson K, Fritzell J. Welfare states and population health: the role of minimum income benefits for mortality. 2014;112:63-71.

156. Neves JA, Vasconcelos FdAGd, Machado ML, Recine E, Garcia GS, Medeiros MATd. The Brazilian cash transfer program (Bolsa Família): A tool for reducing inequalities and achieving social rights in Brazil. 2020:1-17.

157. Nguyen BT, Ford CN, Yaroch AL, Shuval K, Drope J. Food Security and Weight Status in Children: Interactions With Food Assistance Programs. 2017;52(2):S138-S44.

158. Nygaard RM, Marek AP. The Affordable Care Act's Effect on Discharge Disposition of Racial Minority Trauma Patients in the United States. Journal of racial and ethnic health disparities. 2019;6(2):427-35.

159. Okoye OC. Impact of Inclusive Policies on Health and Clinical Outcomes of Noncitizen Migrants: A Systematic Review. 2020.

160. Oliver HC. In the wake of structural adjustment programs: Exploring the relationship between domestic policies and health outcomes in Argentina and Uruguay. Canadian journal of public health = Revue canadienne de sante publique. 2006;97(3):217-21.

161. Osler T, Glance LG, Li W, Buzas JS, Hosmer DW. Survival Rates in Trauma Patients Following Health Care Reform in Massachusetts. JAMA surgery. 2015;150(7):609-15.

162. Owusu-Addo E, Cross R. The impact of conditional cash transfers on child health in low- and middle-income countries: a systematic review. 2014;59(4):609-18.

163. Palmer M. Preconception subsidized insurance: Prenatal care and birth outcomes by race/ethnicity. 2020;29(9):1013-30.

164. Park W, Baek J. The impact of employment protection on health: Evidence from fixed-term contract workers in South Korea. 2019;233:158-70.

165. Pega F, Carter K, Kawachi I, Davis P, Gunasekara FI, Lundberg O, et al. The impact of in-work tax credit for families on self-rated health in adults: a cohort study of 6900 New Zealanders. 2013;67(8):682-8.

166. Pega F, Liu SY, Walter S, Pabayo R, Saith R, Lhachimi SK. Unconditional cash transfers for reducing poverty and vulnerabilities: effect on use of health services and health outcomes in low- and middle-income countries. The Cochrane database of systematic reviews. 2017;11:CD011135.

167. Pettifor A, Wamoyi J, Balvanz P, Gichane MW, Maman S. Cash plus: exploring the mechanisms through which a cash transfer plus financial education programme in Tanzania reduced HIV risk for adolescent girls and young women. 2019;22:e25316-e.

168. Pohl RV, Clark KL, Thomas RC. Minimum wages and healthy diet. 2017.

169. Pons-Vigués M, Diez È, Morrison J, Salas-Nicás S, Hoffmann R, Burstrom B, et al. Social and health policies or interventions to tackle health inequalities in European cities: A scoping review: Accessed 20/10/2016). Available at: <https://www>. ncbi. nlm. nih. gov/pmc …; 2014.

170. Powell-Jackson T, Pereira SK, Dutt V, Tougher S, Haldar K, Kumar P. Cash transfers, maternal depression and emotional well-being: Quasi-experimental evidence from India's Janani Suraksha Yojana programme. Social science & medicine (1982). 2016;162:210-8.

171. Raissian KM, Bullinger LR. Money matters: Does the minimum wage affect child maltreatment rates? 2017;72:60.

172. Rajmil L, Hjern A, Spencer N, Taylor-Robinson D, Gunnlaugsson G, Raat H. Austerity policy and child health in European countries: a systematic literature review. BMC public health. 2020;20(1):564.

173. Reeves A, Clair A, McKee M, Stuckler D. Reductions in the United Kingdom's Government Housing Benefit and Symptoms of Depression in Low-Income Households. American journal of epidemiology. 2016;184(6):421-9.

174. Reeves A, McKee M, Mackenbach J, Whitehead M, Stuckler D. Introduction of a national minimum wage reduced depressive symptoms in low‐wage workers: a quasi‐natural experiment in the UK. 2017;26(5):639-55.

175. Renahy E, Mitchell C, Molnar A, Muntaner C, Ng E, Ali F, et al. Connections between unemployment insurance, poverty and health: a systematic review. 2018;28(2):269.

176. Rispel LC, de Sousa CADP, Molomo BG. Can social inclusion policies reduce health inequalities in sub-Saharan Africa?--A rapid policy appraisal. Journal of health, population, and nutrition. 2009;27(4):492-504.

177. Robinson CA, Zheng X. Household food stamp program participation and childhood obesity. 2011:1.

178. Rubio-Hernandez SP, Ayón C. Pobrecitos los niños: The emotional impact of anti-immigration policies on Latino children. 2016;60:20-6.

179. Sabia JJ. Minimum wages and the economic well‐being of single mothers. 2008;27(4):848.

180. Salinas-Rodrguez A, Torres-Pereda MDP, Manrique-Espinoza B, Moreno-Tamayo K, Sols MMT-R. Impact of the non-contributory social pension program 70 y m \' a s on older adults' mental well-being. 2014;9(11):e113085.

181. Salmasi L, Pieroni L. Immigration policy and birth weight: Positive externalities in Italian law. Journal of health economics. 2015;43:128-39.

182. Salmond C, Crampton P, Atkinson J, Edwards R. A decade of tobacco control efforts in New Zealand (1996-2006): impacts on inequalities in census-derived smoking prevalence. 2012;14(6):664-73.

183. Samari G, Catalano R. The Muslim Ban and preterm birth: Analysis of US vital statistics data from 2009 to 2018. 2020;265:113544.

184. Samuels F, Stavropoulou M. ‘Being able to breathe again': The effects of cash transfer programmes on psychosocial wellbeing. 2016;52(8):1099.

185. Scantlebury RJ, Moody A, Oyebode O, Mindell JS. Has the UK Healthy Start voucher scheme been associated with an increased fruit and vegetable intake among target families? Analysis of Health Survey for England data, 2001–2014. 2018;72(7):623-9.

186. Schillaci MA, Waitzkin H, Sharmen T, Romain SJ. The impact of changing medicaid enrollments on New Mexico's Immunization Program. PloS one. 2008;3(12):e3953.

187. Schmeiser MD. The impact of long-term participation in the supplemental nutrition assistance program on child obesity. 2012;21(4):386-404.

188. Schmitz BAS, Moreira EAM, Freitas MBd, Fiates GMR, Gabriel CG, Fagundes RLM. Public intervention in food and nutrition in Brazil. Archivos latinoamericanos de nutricion. 2011;61(4):361-6.

189. Shahidi FV, Ramraj C, Sod-Erdene O, Hildebrand V, Siddiqi A. The impact of social assistance programs on population health: a systematic review of research in high-income countries. 2019;19(1):1-11.

190. Shahidi FV, Sod-Erdene O, Ramraj C, Hildebrand V, Siddiqi A. Government social assistance programmes are failing to protect the health of low-income populations: evidence from the USA and Canada (2003–2014). 2019;73(3):198-205.

191. Shei A, Costa F, Reis MG, Ko AI. The impact of Brazil's Bolsa Família conditional cash transfer program on children's health care utilization and health outcomes. BMC international health and human rights. 2014;14:10.

192. Siddiqi A, Rajaram A, Miller SP. Do cash transfer programmes yield better health in the first year of life? A systematic review linking low-income/middle-income and high-income contexts. 2018;103(10):920.

193. Silove D, Austin P, Steel Z. No refuge from terror: the impact of detention on the mental health of trauma-affected refugees seeking asylum in Australia. Transcultural psychiatry. 2007;44(3):359-93.

194. Simmons S, Alexander JL, Ewing H, Whetzel S. SNAP participation in preschool-aged children and prevalence of overweight and obesity. 2012;82(12):548-52.

195. Simpson J, Albani V, Bell Z, Bambra C, Brown H. Effects of social security policy reforms on mental health and inequalities: A systematic review of observational studies in high-income countries. 2021:113717-.

196. Slopen N, Fenelon A, Newman S, Boudreaux M. Housing Assistance and Child Health: A Systematic Review. 2018;141(6).

197. Smith-Gagen J, Hollen R, Tashiro S, Cook DM, Yang W. The association of state law to breastfeeding practices in the US. Maternal and child health journal. 2014;18(9):2034-43.

198. Snowden JM, Osmundson SS, Kaufman M, Blauer Peterson C, Kozhimannil KB. Cesarean birth and maternal morbidity among Black women and White women after implementation of a blended payment policy. 2020;55(5):729-40.

199. Sogie-Thomas B, Sankofa J, Reed C, Mfume K, Doamekpor LA. Health Policy Responsiveness: Lessons Learned from Maryland and Prince George's County. Journal of racial and ethnic health disparities. 2018;5(2):366-74.

200. Sommers BD, Blendon RJ, Orav EJ, Epstein AM. Changes in Utilization and Health Among Low-Income Adults After Medicaid Expansion or Expanded Private Insurance. JAMA internal medicine. 2016;176(10):1501-9.

201. Sommers BD, Maylone B, Blendon RJ, Orav EJ, Epstein AM. Three-year impacts of the Affordable Care Act: improved medical care and health among low-income adults. 2017;36(6):1119.

202. Soni A, Wherry LR, Simon KI. How Have ACA Insurance Expansions Affected Health Outcomes? Findings From The Literature: A literature review of the Affordable Care Act's effects on health outcomes for non-elderly adults. 2020;39(3):371.

203. Steel Z, Momartin S, Silove D, Coello M, Aroche J, Tay KW. Two year psychosocial and mental health outcomes for refugees subjected to restrictive or supportive immigration policies. Social science & medicine (1982). 2011;72(7):1149-56.

204. Stehr M. The effect of cigarette taxes on smoking among men and women. 2007;16(12):1333.

205. Stergiopoulos V, Gozdzik A, Misir V, Skosireva A, Sarang A, Connelly J, et al. The effectiveness of a Housing First adaptation for ethnic minority groups: findings of a pragmatic randomized controlled trial. BMC public health. 2016;16(1):1110.

206. Strully KW, Rehkopf DH, Xuan Z. Effects of Prenatal Poverty on Infant Health: State Earned Income Tax Credits and Birth Weight. 2010;75(4):534-62.

207. Studnicki J, Gipson LS, Berndt DJ, Fisher JW, Callandar M, Pracht E, et al. Special healthcare taxing districts: association with population health status. American journal of preventive medicine. 2007;32(2):116-23.

208. Swaminathan S, Sommers BD, Thorsness R, Mehrotra R, Lee Y, Trivedi AN. Association of Medicaid expansion with 1-year mortality among patients with end-stage renal disease. 2018;320(21):2242.

209. Tauras JA. Public policy and smoking cessation among young adults in the United States. 2004;68(3):321.

210. Taylor CJ. Health consequences of laws and public policies that target, or protect, marginalized populations. 2020;14(2):e12753.

211. Terry-McElrath YM, O'Malley PM, Johnston LD. Foods and beverages offered in US public secondary schools through the National School Lunch Program from 2011–2013: early evidence of improved nutrition and reduced disparities. 2015;78:52-8.

212. Tipirneni R, Kullgren JT, Ayanian JZ, Kieffer EC, Rosland A-M, Chang T, et al. Changes in Health and Ability to Work Among Medicaid Expansion Enrollees: a Mixed Methods Study. Journal of general internal medicine. 2019;34(2):272-80.

213. Trivedi AN, Bailie R, Bailie J, Brown A, Kelaher M. Hospitalizations for Chronic Conditions Among Indigenous Australians After Medication Copayment Reductions: the Closing the Gap Copayment Incentive. Journal of general internal medicine. 2017;32(5):501-7.

214. Uchimura K, Ngamvithayapong-Yanai J, Kawatsu L, Ohkado A, Yoshiyama T, Ito K, et al. Permanent employment or public assistance may increase tuberculosis survival among working-age patients in Japan. The international journal of tuberculosis and lung disease : the official journal of the International Union against Tuberculosis and Lung Disease. 2015;19(3):312-8.

215. Valdovinos EM, Niedzwiecki MJ, Guo J, Hsia RY. The association of Medicaid expansion and racial/ethnic inequities in access, treatment, and outcomes for patients with acute myocardial infarction. 2020;15(11):e0241785.

216. Van Dyke ME, Komro KA, Shah MP, Livingston MD, Kramer MR. State-level minimum wage and heart disease death rates in the United States, 1980-2015: A novel application of marginal structural modeling. 2018;112:97-103.

217. Ver Ploeg M, Mancino L, Lin BH, Guthrie J. US Food assistance programs and trends in children's weight. 2008;3(1):22-30.

218. Vernice NA, Pereira NM, Wang A, Demetres M, Adams LV. The adverse health effects of punitive immigrant policies in the United States: A systematic review. 2020;15(12):e0244054-e.

219. Wadhera RK, Joynt Maddox KE, Fonarow GC, Zhao X, Heidenreich PA, DeVore AD, et al. Association of the Affordable Care Act's Medicaid Expansion With Care Quality and Outcomes for Low-Income Patients Hospitalized With Heart Failure. Circulation Cardiovascular quality and outcomes. 2018;11(7):e004729.

220. Waldfogel J. Welfare reforms and child well-being in the US and UK. 2007.

221. Walter S, Glymour M, Avendano M. The Health Effects of US Unemployment Insurance Policy: Does Income from Unemployment Benefits Prevent. 2014.

222. Wherry LR, Fabi R, Schickedanz A, Saloner B. State And Federal Coverage For Pregnant Immigrants: Prenatal Care Increased, No Change Detected For Infant Health. Health affairs (Project Hope). 2017;36(4):607-15.

223. Wickham S, Bentley L, Rose T, Whitehead M, Taylor-Robinson D, Barr B. Effects on mental health of a UK welfare reform, Universal Credit: a longitudinal controlled study. 2020;5(3):e157-e64.

224. Wicks-Lim J, Arno PS. Improving population health by reducing poverty: New York’s earned income tax credit. 2017;3:373-81.

225. Wiggins A, Karaye IM, Horney JA. Medicaid expansion and infant mortality, revisited: A difference-in-differences analysis. 2020;55(3):393-8.

226. Wilde ET, Rosen Z, Couch K, Muennig PA. Impact of welfare reform on mortality: an evaluation of the Connecticut jobs first program, a randomized controlled trial. American journal of public health. 2014;104(3):534-8.

227. Wilde PE, Conrad Z, Rehm CD, Pomeranz JL, Penalvo JL, Cudhea F, et al. Reductions in national cardiometabolic mortality achievable by food price changes according to Supplemental Nutrition Assistance Program (SNAP) eligibility and participation. 2018;72(9):817-24.

228. Winkelman TNA, Chang VW. Medicaid expansion, mental health, and access to care among childless adults with and without chronic conditions. 2018;33(3):376.

229. Wolf DA, Monnat SM, Montez JK. Effects of US state preemption laws on infant mortality. 2021;145:106417.

230. Ydreborg B, Ekberg K, Nordlund A. Health, quality of life, social network and use of health care: a comparison between those granted and those not granted disability pensions. Disability and rehabilitation. 2006;28(1):25-32.

231. Zhang W, Wu Q. The Relationship Between Public Sector Employment and Population Health: Evidence From the 1980s and Its Contemporary Implications. International journal of health services : planning, administration, evaluation. 2019;49(3):555-81.

232. Hamad R, Collin DF, Rehkopf DH. Estimating the short-term effects of the earned income tax credit on child health. 2018;187(12):2633-41.

233. Hamad R, Rehkopf DH. Poverty, pregnancy, and birth outcomes: a study of the earned income tax credit. 2015;29(5):444-52.

234. Herd P, Schoeni RF, House JS. Does the Supplemental Security Income program reduce disability among the elderly. 2005.

235. Lenhart O. The effects of income on health: new evidence from the Earned Income Tax Credit. 2019;17(2):377-410.

236. Lenhart O. The effects of state‐level earned income tax credits on suicides. 2019;28(12):1476-82.

237. Pega F. The effect of anti-poverty and in-work tax credits for families on self-rated health in parents in New Zealand.349.

238. Pega F, Carter K, Blakely T, Lucas PJ. In‐work tax credits for families and their impact on health status in adults. 2013(8).

239. Scantlebury R, Moody A, Oyebode O, Mindell J. OP19 Has the UK Healthy Start voucher scheme been associated with an increased fruit and vegetable intake amongst target families? Analysis of Health Survey for England data, 2001–2014: BMJ Publishing Group Ltd; 2016.

240. Tessler RA, Mooney SJ, Quistberg DA, Rowhani-Rahbar A, Vavilala MS, Rivara FP. State-Level Beer Excise Tax and Firearm Homicide in Adolescents and Young Adults. 2019;56(5):708-15.
